# Phosphorylation and Internalization of Lysophosphatidic Acid Receptors LPA_1_, LPA_2_, and LPA_3_

**DOI:** 10.1371/journal.pone.0140583

**Published:** 2015-10-16

**Authors:** Rocío Alcántara-Hernández, Aurelio Hernández-Méndez, Gisselle A. Campos-Martínez, Aldo Meizoso-Huesca, J. Adolfo García-Sáinz

**Affiliations:** Instituto de Fisiología Celular, Universidad Nacional Autónoma de México, Apartado Postal 70–248, México D.F., México 04510; University of Oldenburg, GERMANY

## Abstract

**Results:**

The lysophosphatidic acid receptors LPA_1_, LPA_2_, and LPA_3_ were individually expressed in C9 cells and their signaling and regulation were studied. Agonist-activation increases intracellular calcium concentration in a concentration-dependent fashion. Phorbol myristate acetate markedly inhibited LPA_1_- and LPA_3_-mediated effect, whereas that mediated by LPA_2_ was only partially diminished; the actions of the phorbol ester were inhibited by bisindolylmaleimide I and by overnight incubation with the protein kinase C activator, which leads to down regulation of this protein kinase. Homologous desensitization was also observed for the three LPA receptors studied, with that of LPA_2_ receptors being consistently of lesser magnitude; neither inhibition nor down-regulation of protein kinase C exerted any effect on homologous desensitization. Activation of LPA_1–3_ receptors induced ERK 1/2 phosphorylation; this effect was markedly attenuated by inhibition of epidermal growth factor receptor tyrosine kinase activity, suggesting growth factor receptor transactivation in this effect. Lysophosphatidic acid and phorbol myristate acetate were able to induce LPA_1–3_ phosphorylation, in time- and concentration-dependent fashions. It was also clearly observed that agonists and protein kinase C activation induced internalization of these receptors. Phosphorylation of the LPA_2_ subtype required larger concentrations of these agents and its internalization was less intense than that of the other subtypes.

**Conclusion:**

Our data show that these three LPA receptors are phosphoproteins whose phosphorylation state is modulated by agonist-stimulation and protein kinase C-activation and that differences in regulation and cellular localization exist, among the subtypes.

## Introduction

Lysophosphatidic acid (LPA) is one of the so-called, “bioactive lipids”, that participates not only in cell metabolism but also as an autacoid or local hormone, communicating cells. LPA is involved in a very large number of physiological processes, modulating the function of many organs and systems (gastrointestinal apparatus, nervous, immune, and urogenital systems, and others); this lipid takes part in embryonic development and also has a “dark side” being involved in the pathogenesis of diseases (fibrosis, inflammation, and cancer, among many others); at the cellular level, it modulates migration, chemotaxis, proliferation, survival, and other processes (see [1–5] and references therein). LPA actions are mainly exerted through a family of G protein-coupled receptors (GPCRs), that is, the LPA receptors, comprising six members that are currently designated LPA_1–6_; the possibility that GPR87 could be also a member of this family has been suggested, i. e., such as LPA_7_ [1–6]. Of these receptors, LPA_1_, LPA_2_ and LPA_3_ are phylogenetically related among themselves and also with those of other bioactive lipids (the endothelial differentiation gene [“edg”] family); the remaining LPA receptors are distant phylogenetically from these and are more closely related with the purinergic receptor family [1–7]. Evolutionary aspects of these receptors, among vertebrates, have been recently reported [7]. It is also known that LPA can modulate transcription through nuclear receptors, such as the peroxisome proliferator-activated receptor γ [8]. LPA also activates the TRPV1 ion channel involved in the control of body temperature and nociception [9].

Our present work deals exclusively with the LPA_1–3_ receptors. The actions of these receptors have been studied using many different natural (i. e. endogenously expressed) and transfected cellular and systemic models. However, few studies have analyzed LPA_1–3_ desensitization and internalization employing the same cellular model. In particular, the phosphorylation of these receptors has been scarcely studied. To the best of our knowledge, solely LPA_1_ receptor phosphorylation has been reported and only by our own group [4, 10–14]. The present work was designed to fulfill this gap in knowledge.

Desensitization, defined as a stage of reduced sensitivity to a particular stimulus, can involve a large number of processes with different time scales. It is generally accepted that GPCR sensitivity (desensitization/ resensitization) involves phosphorylation/ dephosphorylation cycles controlled by particular protein kinases and phosphatases [15–20]; although there is evidence also for phosphorylation-independent desensitization [21]. The majority of current data indicate that agonist-induced receptor desensitization (homologous desensitization) involves receptor phosphorylation by G protein-coupled receptor kinases (GRKs) whereas desensitization of unoccupied receptors, i. e. agonist-independent (heterologous desensitization) mainly involves signaling activated kinases such as the second messenger-activated kinases, protein kinase A and protein kinase C (PKC), among others [15–20]. Receptor internalization appears to be related with receptor phosphorylation. Current ideas indicate that phosphorylated receptors interact with β-arrestins and act as molecular bridges with clathrin, clustering receptors that internalize in coated vesicles; such internalization can lead receptors to plasma membrane recycling, trafficking to other compartments or to degradation. Variation in the phosphorylation pattern of a given receptor has been observed and it has been suggested that such phosphorylation “bar code” might determine receptor’s destination and function [19, 22, 23]. Recently, we reported differential association of α_1B_-adrenergic receptors to Rab proteins during internalizations induced by agonists (homologous) or unrelated (heterologous) stimuli [24].

*In silico* analysis showed that these three receptors, i. e., LPA_1_, LPA_2_, and LPA_3_, possess putative phosphorylation sites for a variety of protein kinases, particularly GRKs and PKC isoforms, with marked differences among them [4]. These receptors fused to the enhanced green fluorescent protein (eGFP) were expressed in C9 cells and their signaling, sensitivity to PKC activation, phosphorylation, and internalization were studied comparatively. Our results clearly indicate that LPA_1_, LPA_2,_ and LPA_3_ receptors are phosphorylated and internalize in response to LPA and PKC activation. Differences in agonist sensitivity and degree of internalization/ desensitization were also observed.

## Materials and Methods

### 1. Materials

LPA (oleyl-sn-glycerol 3-phosphate), phorbol myristate acetate (PMA), G418, Ham’s F12 Kaighn’s modification medium, protease inhibitors and DNA purification kits were purchased from Sigma Chemical Co. Phosphate-free Dulbecco’s modified Eagle’s medium, fetal bovine serum, trypsin, antibiotics, and other reagents used for cell culture were from Life Technologies. Fura-2 AM was obtained from Invitrogen and agarose-coupled protein A, from Upstate Biotechnology. Bisindolylmaleimide I and AG1478 were purchased from Calbiochem whereas EGF was obtained from Preprotech. [^32^P]Pi (8,500–9,120 Ci/mmol) was obtained from Perkin Elmer Life Sciences. The plasmid construction for the expression of mouse LPA_1_ receptor fused to the eGFP was previously described [11] and those used for the expression of human LPA_2_ and LPA_3_, also fused to the eGFP at the carboxyl termini, were obtained from GeneCopoeia and OriGene, respectively. Rabbit polyclonal antibodies against ERK 1/2 and phospho-ERK 1/2 were from Cell Signaling Technology. Secondary antibodies were obtained from Zymed. For Western blotting an anti-GFP monoclonal antibody from Clontech was employed. Rabbit antisera against eGFP were generated at our laboratory using standard procedures [25] by immunizing New Zealand rabbits with *E*. *coli*-overespressed GST-fused eGFP (1 mg/ kg, each 2 weeks for at least 6 times) and have been previously characterized and compared with commercial antibodies [11, 12, 14]. Animals were handled and maintained in individual cages, with free access to water and rabbit chow, in rooms with controlled air temperature and lighting (12 h/12 h); handling and bleeding (marginal ear vein) were performed under the direct supervision of one of the Veterinary Doctors in change of the animal facility.

### 2. Cell culture and transfection

C9 cells (Clone 9, rat hepatic epithelial cells, CRL-1439™), obtained directly from American Type Culture Collection, were cultured in Ham’s F12 Kaighn’s modification medium supplemented with 10% fetal bovine serum, 100 μg/ml streptomycin, 100 units/ml penicillin and 0.25 μg/ml amphotericin B at 37°C under a 95% air and 5% CO_2_ atmosphere, as described previously [11]. The medium was replaced with one containing 1% fetal bovine serum, 12–16 h before the experiment. C9 cells stably expressing the mouse LPA_1_ receptor fused at the carboxyl termini with eGFP were those previously described [11]. Stable expression of the human LPA_2_ and LPA_3_ receptors fused to eGFP was obtained by transfecting wild type C9 cells with the plasmid constructs described above using lipofectamine 2000, following the manufacturer’s instructions; cells were transfected three times to increase the gene transfer efficiency [26]. Clones expressing these constructs were selected by resistance to incubation with G418 (600 ng/ml) and by their robustness of response to LPA (increase in intracellular calcium concentration). After this selection (3 cell passages) the cells were cultured in media containing 300 ng/ml of G418. As depicted in”Fig A in S1 File”, expression of LPA_1_ and LPA_2_ receptors was similar (~80%) whereas that of LPA_3_ was consistently lower (~60%), as evidenced by fluorescence imaging and flow cytometry. Flow cytometry was performed using an Attune NxT Acoustic Focusing Cytometer, employing excitation of 488 nm; data were analyzed using the Attune cytometric software included with the equipment.

### 3. Intracellular calcium concentration ([Ca^2+^]_i_)

This procedure has been described previously [11]. Briefly, cells were loaded with 2.5 μM Fura-2 AM in Krebs–Ringer–Hepes containing 0.05% bovine serum albumin (pH 7.4) for 1 h at 37°C and then washed to eliminate unincorporated indicator. Determinations were carried in an AMINCO-Bowman Series 2 luminescence spectrometer, employing 340 and 380 nm excitation wavelengths and an emission wavelength of 510 nm; chopper interval was 0.5 sec. The intracellular calcium concentration was calculated as described by Grynkiewicz et al. [27]. In the experiments where prolonged incubation was required (resensitization studies), cells were stimulated, washed, and the incubation was continued in the presence of Fura-2 AM, to avoid dye depletion. Cells were washed again to remove extracellular fluorescent dye, and determinations made as indicated above; this procedure did not alter the magnitude of the control calcium responses.

### 4. ERK 1/2 phosphorylation

Cells were cultured to near confluence in 6 well plates and stimulated with the agents tested for the times indicated. Total cellular extracts were obtained by lysing the cells in Laemmli’s sample buffer containing 5% β-mercaptoethanol. The cell extracts were subject to 10% SDS-PAGE and transferred to nitrocellulose membranes. Membranes from samples obtained in parallel were incubated overnight at 4°C, with anti-pERK 1/2 (1:2000) or anti ERK 1/2 (1:2000). The membranes were washed and incubated for 1 h at room temperature with a horseradish peroxidase-conjugated secondary antibody (1:10,000) for enhanced chemiluminescence detection.

### 5. Phosphorylation of LPA_1–3_ receptors

The procedure was very similar to that previously described to study LPA_1_ receptor phosphorylation [11, 12, 14]. In brief, cells were incubated in phosphate-free Dulbecco’s modified Eagle’s medium containing 100 μCi/ml [^32^P]Pi for 3 h at 37°C. LPA_1_- and LPA_2_-expressing cells were cultured in 6 well plates, whereas those expressing LPA_3_ receptors were cultured in 10 cm dishes. Labeled cells were stimulated with the agents tested and then washed twice with ice-cold phosphate buffered saline and solubilized in buffer containing 10 mM Tris, 150 mM NaCl, 1% sodium cholate, 1% Nonident P40, protease and phosphatase inhibitors, pH 7.4 [11, 12, 14]. The lysates were incubated overnight with protein A-agarose and anti-GFP serum with constant agitation at 4°C. Samples were washed and the pellets containing the immunocomplexes were solubilized in Laemmli’s sample buffer containing 5% β-mercaptoethanol. Proteins were separated using 10% SDS-PAGE and electrotransferred onto polyvinyliden fluoride membranes. Receptor phosphorylation was analyzed with a Molecular Dynamics Typhoon PhosphorImager and the ImageJ software (http://rsb.info.nih.gov/ij/).

### 6. Receptor internalization- Imaging

Cells were seeded at approximately 30% confluence onto glass-bottomed Petri dishes and cultured for 3 h at 37°C in media containing 1% serum. After treatment, cells were washed three times with phosphate buffered saline and fixed with 4% paraformaldehyde in 0.1 M phosphate buffer for 20 min at room temperature; samples were then washed three additional times with phosphate buffered saline. The fluorescent images were acquired with an Olympus Fluoview FV10 confocal microscope with a water-immersion objective (60X). To determine receptor internalization, the plasma membrane was delineated utilizing the differential interference contrast imaging, and fluorescence in this region was quantified employing the ImageJ software. At least 5 or 6 images of different cultures were taken for each condition. Data were normalized as follows: for each experiment, fluorescence (arbitrary units) at the plasma membrane of baseline samples were pooled and the average was considered as 100%.

### 7. Statistical Analysis

EC_50_ and IC_50_ values were calculated from the individual concentration-response curves employing the software included in the GraphPad Prism 6 program and reported as the rounded range of values observed. Similarly, analysis of variance with the Bonferroni’s post-test was performed using the statistical software included in the Prism 6 program. A *p* value < 0.05 was considered statistically significant.

### 8. Ethics statement

This study was carried out in strict accordance with the recommendations in the Guide for the Care and Use of Laboratory Animals of the National Institutes of Health and the Mexican Laws and Regulations on this matter. Protocol AGS29-14 was approved to our work by the Institutional Committee for the use of laboratory animals, Instituto de Fisiología Celular.

## Results

**Fig 1:**
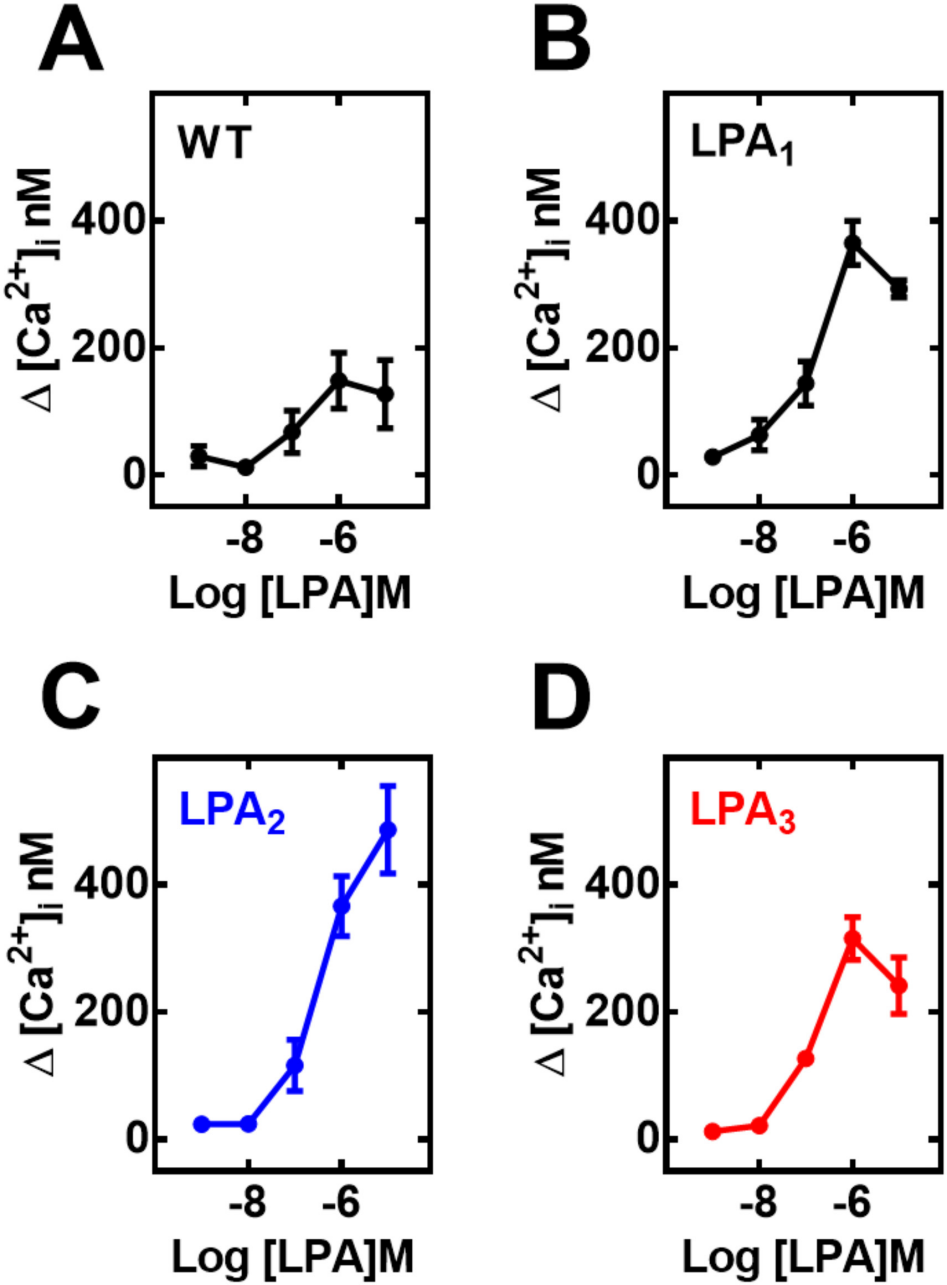
Effect of LPA on intracellular calcium concentration ([Ca^2+^]_i_). Wild type C9 cells (panel A) or overexpressing LPA_1_ (panel B), LPA_2_ (panel C) or LPA_3_ (panel D) were stimulated by different concentrations of LPA. Plotted are the increases in intracellular calcium as mean ± S. E. M. of 4–5 experiments using different cell preparations.

In agreement with previous results [10, 11] wild-type C9 cells increase intracellular calcium concentration in response to LPA; this is likely due to the activation of LPA_1_ and LPA_2_ that are expressed in these cells [11]. The effect was concentration-dependent with a maximal calcium concentration increase of ~150–200 nM and EC_50_ values in the range of ~ 200–400 nM (Fig 1, panel A). Expression of LPA_1–3_ receptors markedly augmented this calcium response to increases the cation’s concentration to ~ 300 nM (in cells over-expressing LPA_3_ receptors) and to 450–600 nM (cells over-expressing LPA_1_ or LPA_2_ receptors); concentration-response curves yielded EC_50_ values in the range of 200–400 nM in LPA_1_- or LPA_3_- overexpressing cells. In cells overexpressing LPA_2_, the LPA concentration response curve was slightly, but consistently, shifted to the right and no clear saturation was achieved at the concentrations tested (Fig 1, panels B-D; representative images in “Fig B in S1 File”).

**Fig 2:**
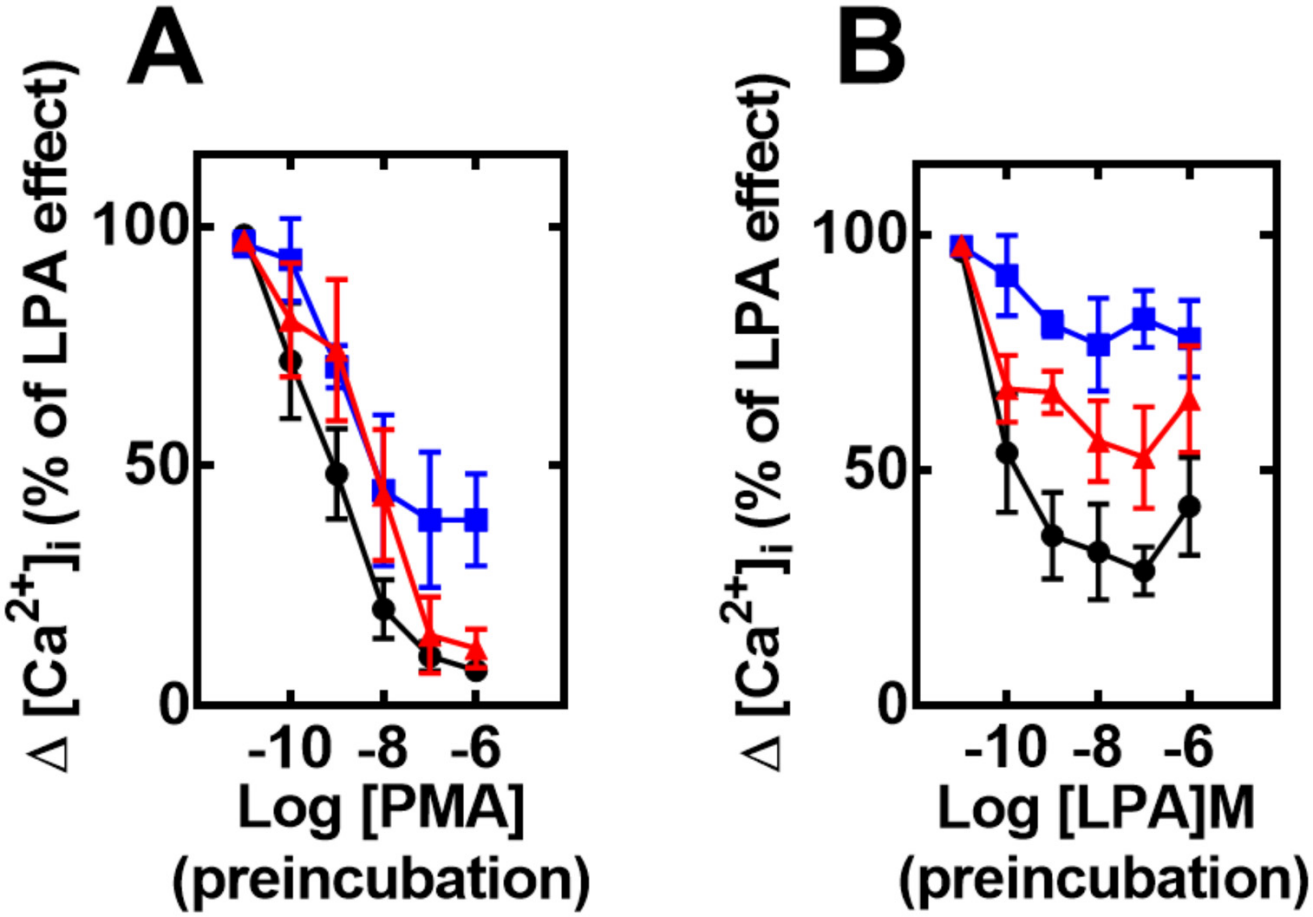
Effect of preincubation with PMA (heterologous desensitization) or LPA (homologous desensitization) on LPA-induced intracellular calcium concentration ([Ca^2+^]_i_). Cells overexpressing LPA_1_ (black, circles), LPA_2_ (blue, squares) or LPA_3_ (red, triangles) receptors were preincubated in the absence or presence of different PMA concentrations for 2 min and then challenged with 1 μM LPA (panel A) or with different concentrations of LPA for 10 minutes, washed 3 times and then challenged with 1 μM LPA (panel B) and the increase in intracellular free calcium concentration was determined. Plotted are the increases in calcium as the percentage of that obtained in cells preincubated without any agent as mean ± S. E. M. of 6 experiments using different cell preparations.

**Fig 3:**
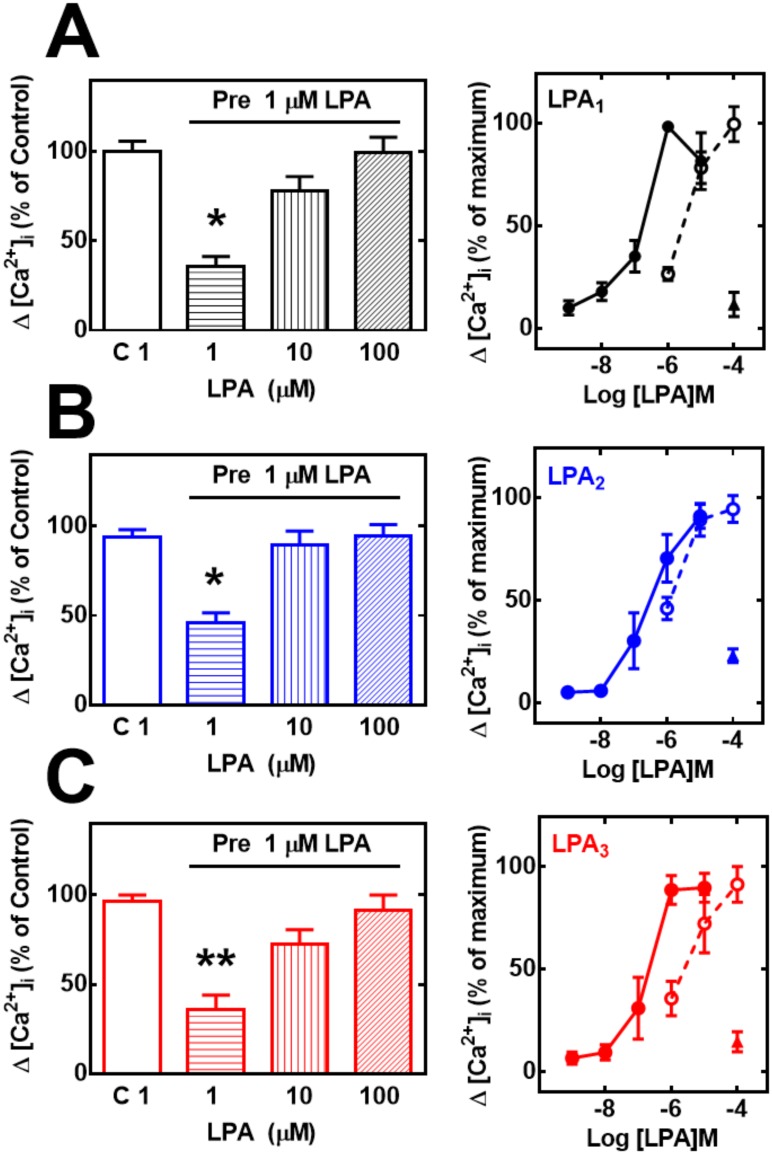
Effect of preincubation with PMA (heterologous desensitization) or LPA (homologous desensitization) on LPA-induced intracellular calcium concentration ([Ca^2+^]_i_) using high agonist concentrations. Cells overexpressing LPA_1_ (panel A, black), LPA_2_ (panel B, blue) or LPA_3_ (panel C, red) receptors were preincubated in the absence or presence of 1 μM LPA for 10 min, extensively washed, then challenged with the indicated concentrations of LPA, and the increase in intracellular free calcium concentration was determined. Plotted are the increases in calcium as the percentage of that obtained in cells preincubated without any agent and challenged with 1 μM LPA (C 1 in the abscisa) (% of control) as mean ± S. E. M. of 6 experiments using different cell preparations. In the right panels concentration response curves are plotted. Data from Fig 1 (without pre-stimulation) were normalized and re-plotted as percentage of the maximal response (solid symbols and continuous connecting lines) together with those in the left panels of this figure, normalized in the same way (open symbols, dotted connected lines). The response of cells preincubated with 1 μM PMA for 2 min, washed, and then challenged with to 100 μM LPA is also presented (solid triangles).

In order to evaluate heterologous desensitization (PMA-induced) cells were incubated with different concentrations of PMA for 2 min and then challenged with 1 μM LPA. As illustrated in Fig 2 (panel A), cells overexpressing LPA_1_ receptors were very sensitive to PMA (IC_50_ 1–3 nM) whereas those overexpressing LPA_2_ and LPA_3_ receptors were slightly less sensitive (IC_50_ values in the range of 3–10 nM). Interestingly, in LPA_2-_overexpressing cells PMA was unable to completely blunt the calcium response to the bioactive lipid, i. e., a remaining ~ 30–40% response was consistently observed, even at the highest PMA concentration tested (Fig 2, panel A; representative tracings are presented in “Fig B in S1 File”). In the case of LPA-induced (homologous) desensitization, cells expressing the different LPA receptors were incubated for 10 min in the presence of different LPA concentrations; after this, the cells were washed twice to remove LPA and were then challenged with 1 μM LPA. The results showed that cells expressing any of the three receptors were affected by the preincubation, even at very low concentrations of the agonist (Fig 2, panel B); the preincubation and washing procedures were not responsible for this as evidenced by the control responses (baseline, vehicle during the preincubation). Agonists-mediated decreases occurred in a concentration-dependent fashion, with a maximum at the concentration of ~ 100 nM. Interestingly, the magnitude of this desensitization process was LPA_1_ ≥LPA_3_> LPA_2_ (Fig 2, panel B). When cells were incubated for 10 min with 1 μM LPA, washed as indicated above and then challenged with 1–100 μM LPA the calcium response increased. This indicated that rather than decreasing the maximal response, homologous desensitization reduced the cell’s sensitivity to LPA (Fig 3, left panels). This was more clearly shown when the concentration-response curves to LPA in control and agonist-pretreated cells were normalized and plotted (Fig 3, right panels). It is worth noticing that the shift in the curves was more pronounced in LPA_1_- or LPA_3_-expressing cells, than in those that expressed the LPA_2_ subtype. The response to 100 μM LPA of PMA-treated cells was very small (Fig 3, right panels).

**Fig 4:**
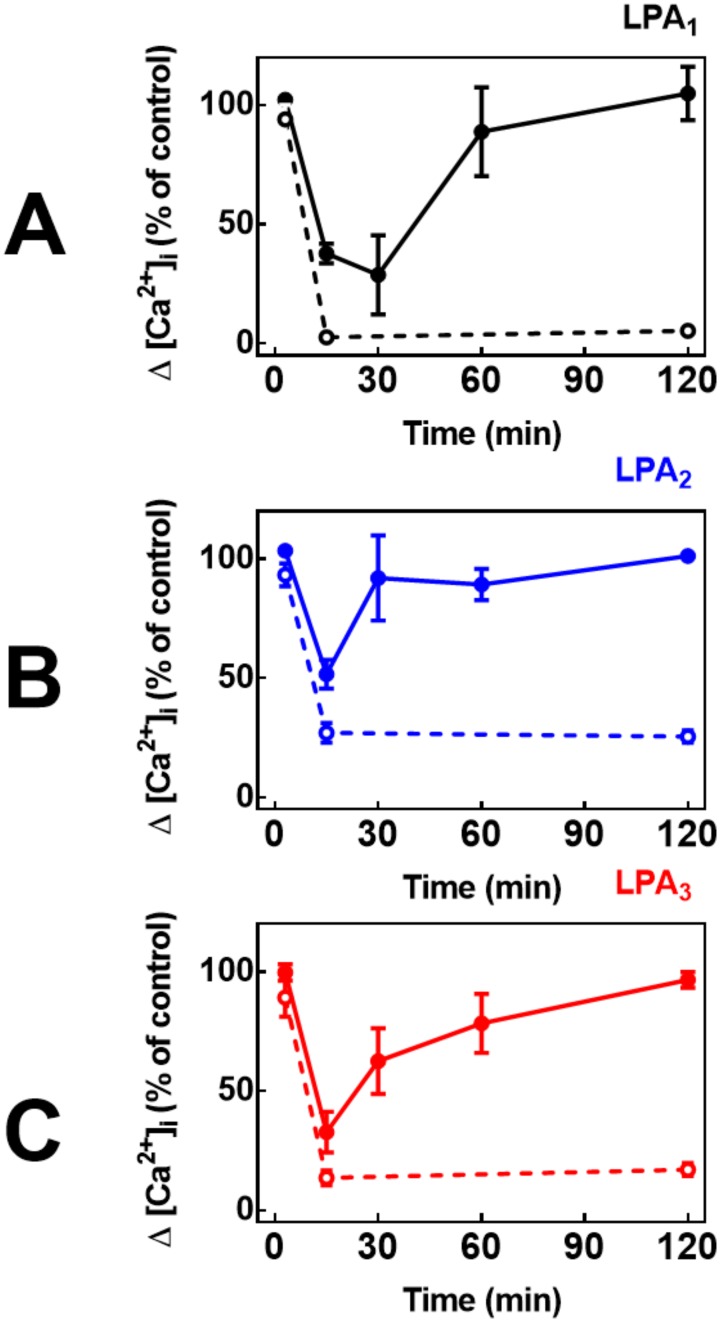
Reversibility (homologous) and persistency (heterologous) of the desensitizations of the intracellular calcium response to LPA. Cells overexpressing LPA_1_ (panel A, black symbols and lines), LPA_2_ (panel B, blue symbols and lines) or LPA_3_ (panel C, red symbols and lines) receptors were preincubated in the presence of 1 μM PMA for 2 min (open symbols, dotted lines) or with 1 μM LPA for 10 minutes and then extensively washed. Incubation was continued for the times indicated and cells were challenged with 1 μM LPA and the increase in intracellular free calcium concentration was determined. Plotted are the increases in calcium as the percentage of that obtained in cells preincubated without any agent (control, time 0) as mean ± S. E. M. of 5–6 experiments using different cell preparations.

The possibility that the cell responsiveness to LPA could resensitize was considered. To study this, cells were incubated with 1 μM PMA for 2 min or with 1 μM LPA for 10 min and then extensively washed. After this procedure, the incubation continued for the times indicated (up to 2 h) and then cells were challenged with 1 μM LPA. As shown in Fig 4 the cell responsiveness to LPA recover after washing, during the subsequent incubation but not when the active phorbol ester, PMA, was employed. These resensitization patterns were similar for the three LPA receptor subtypes studied (Fig 4). The resenstization time-courses after homologous desensitization were inversely correlated with the desensitization magnitudes, i. e., cells expressing LPA_2._receptors resensitize clearly faster that those expressing the LPA_1_ or LPA_3_ subtypes (Fig 4). We were unable to detect changes in receptor density (degradation) in response to LPA or PMA during these incubations times, as evidenced by anti-eGFP Western blotting of extracts from cells incubated in the presence cycloheximide (50 μM) to prevent new protein synthesis; cycloheximide was added 30 min before addition of LPA or PMA and was present during the whole incubation period (“Fig C in S1 File”).

**Fig 5:**
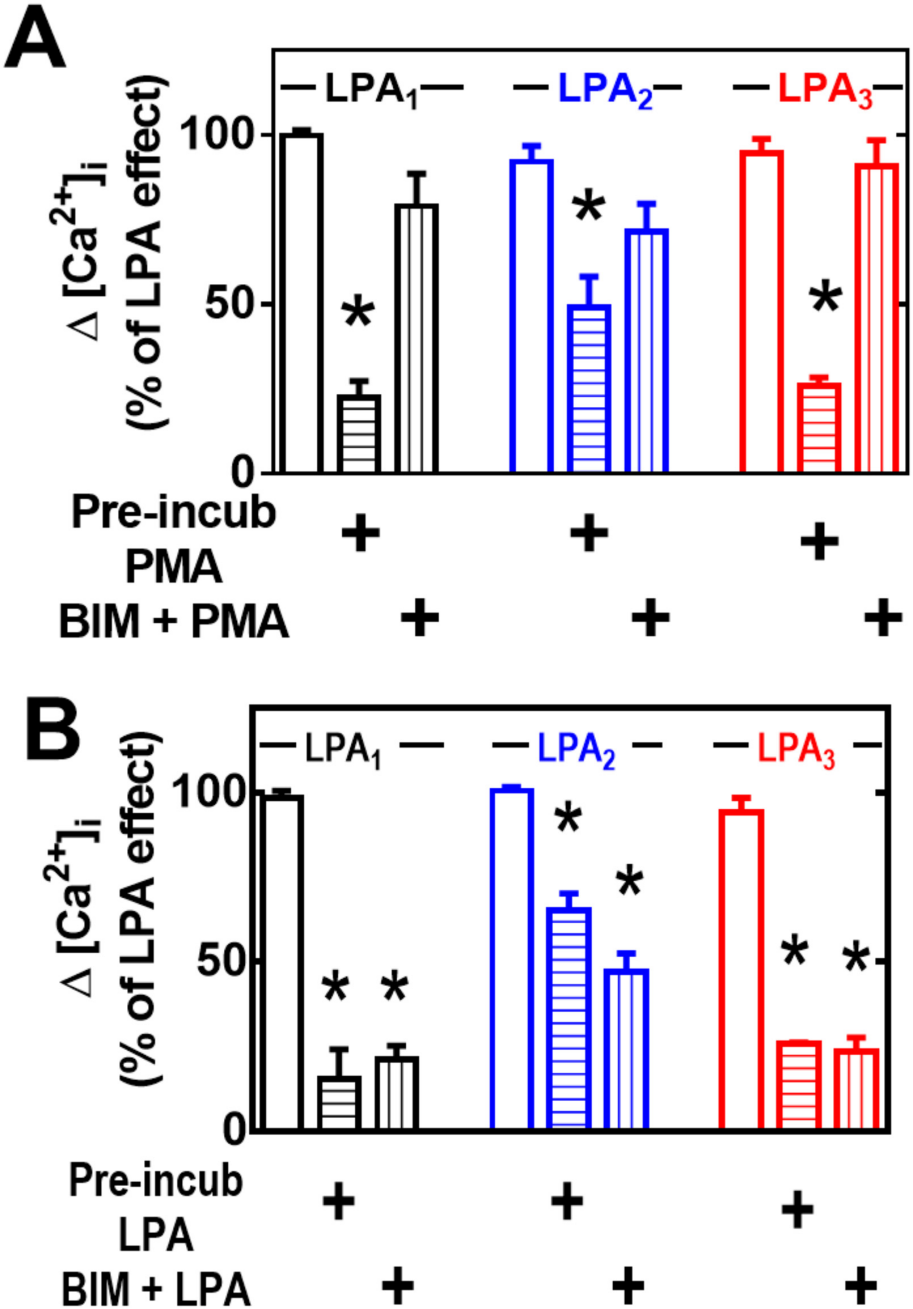
Effect of PKC inhibition on heterologous (PMA-induced) or homologous (LPA-induced) desensitization. Cells overexpressing LPA_1–3_ receptors were preincubated for 15 min in the presence of the PKC inhibitor, bisindolylmaleimide I (BIM), and then subjected to the desensitization protocols (indicated under the Experimental section and in Fig 2), using 1 μM PMA or 1 μM LPA. Cells were challenged with 1 μM LPA and the increase in intracellular free calcium concentration was determined. Plotted are the increases in calcium as the percentage of that obtained in cells preincubated without any agent as mean ± S. E. M. of 6–8 experiments using different cell preparations. *p < 0.001 vs. baseline.

The PKC inhibitor, bisindolylmaleimide I, markedly diminished PMA-induced desensitization in cells expressing any of the LPA receptor subtypes (Fig 5, panel A) but was unable to alter LPA-induced desensitization (Fig 5, panel B).

**Fig 6:**
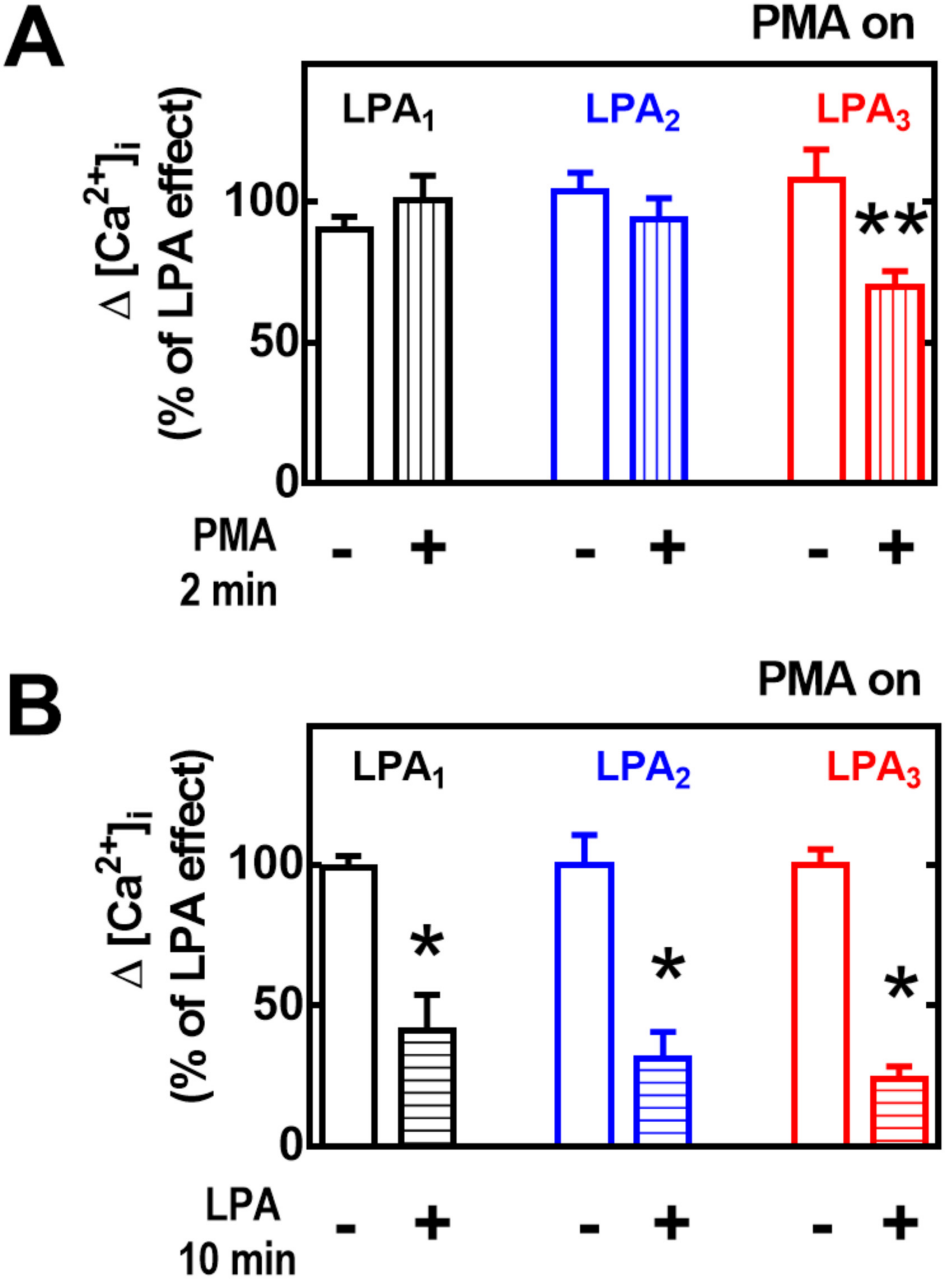
Effect of PKC down regulation on heterologous (PMA-induced) or homologous (LPA-induced) desensitization. Cells overexpressing LPA_1–3_ receptors were preincubated overnight with 1 μM PMA, and then subjected to the desensitization protocols (indicated under the Experimental section and in Fig 2) using 1 μM PMA (2 min) or 1 μM LPA (10 min). Cells were challenged with 1 μM LPA, and the increase in intracellular free calcium concentration was determined. Plotted are the increases in calcium as the percentage of that obtained in cells preincubated without any agent as mean ± S. E. M. of 5–7 experiments using different cell preparations. *p < 0.001 vs baseline; **p <0.05 vs baseline.

It is well-known that over-night treatment with 1 μM PMA markedly down-regulates conventional and novel PKC isoforms [14, 28]. Consistent with the previous data, this treatment markedly reduced or abolished PMA-induced desensitization (Fig 6, panel A) but was completely unable to alter agonist-induced desensitization (Fig 6, panel B).

**Fig 7:**
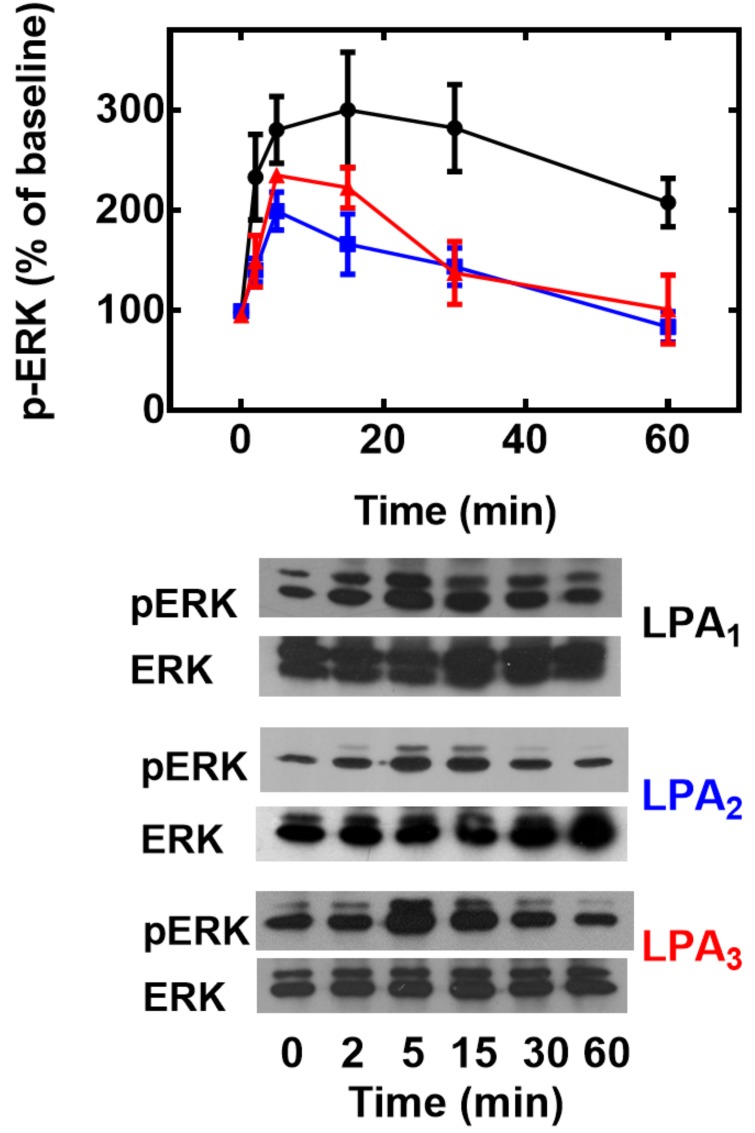
Effect of LPA on ERK 1/2 phosphorylation. Cells overexpressing LPA_1_ (black, circles), LPA_2_ (blue, squares) or LPA_3_ (red, triangles) receptors were incubated for the times indicated in the presence of 1 μM LPA, incubation was terminated and phospho-ERK 1/2 (pERK) and total ERK 1/2 (ERK) were assayed by Western blotting. Plotted are the increases in phospho-ERK 1/2 as mean ± S. E. M. of 4–5 experiments using different cell preparations. Representative Western blots are presented for the different receptor subtypes.

**Fig 8:**
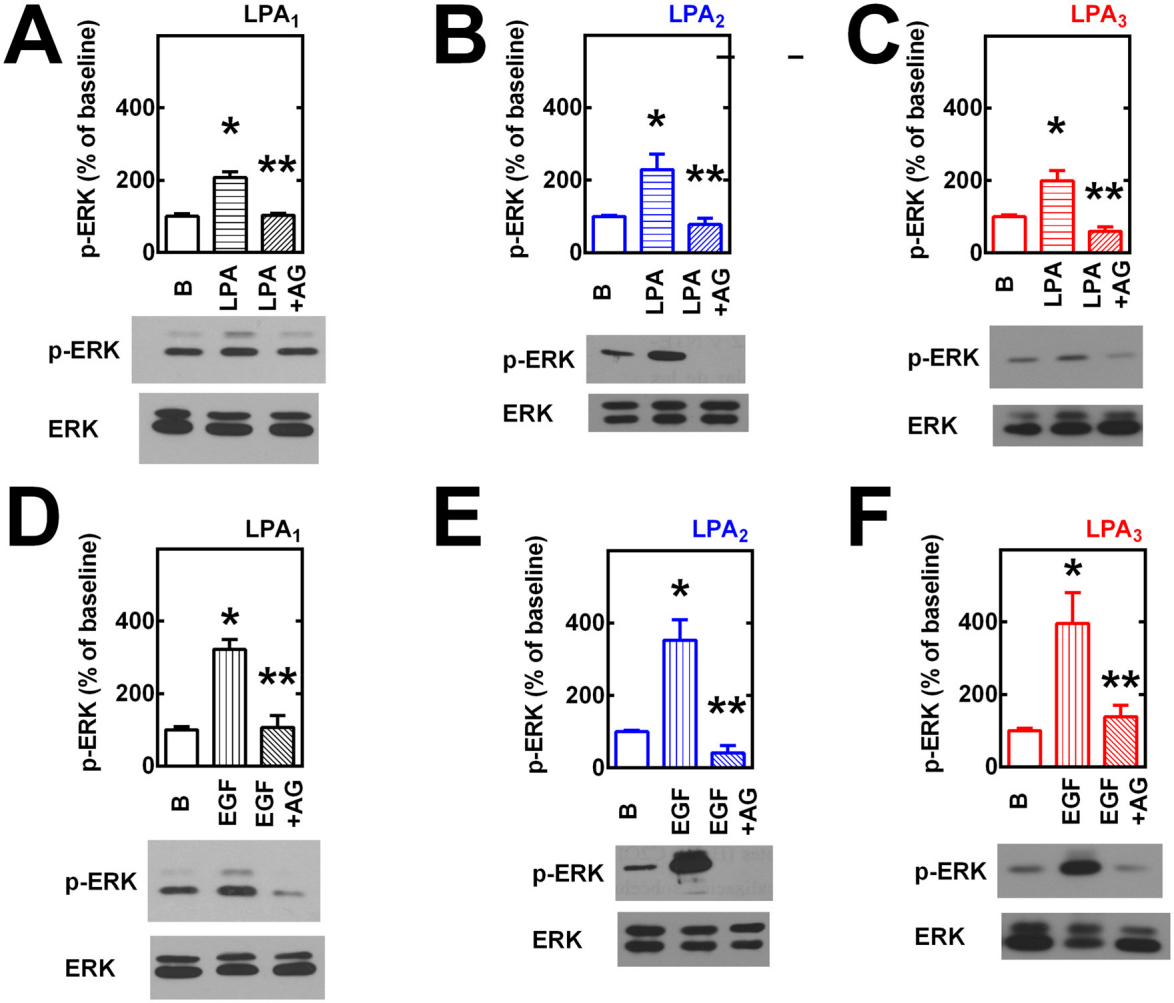
Transactivation of EGF receptors in LPA-induced ERK 1/2 phosphorylation. Cells overexpressing LPA_1_ (panels A and D), LPA_2_ (panels B and E) or LPA_3_ (panels C and F) receptors were incubated in the absence or presence of 10 μM AG1478 (AG) for 30 min and then challenged with 1 μM LPA (panels A-C) or 100 ng/ml EGF (panels D-F) for 5 min; incubation was terminated and phospho-ERK 1/2 (pERK) and total ERK 1/2 (ERK) were assayed by Western blotting. Plotted are the increases in phospho-ERK 1/2 as mean ± S. E. M. of 4–5 experiments using different cell preparations. Representative Western blots are presented for the different receptor subtypes. *p < 0.001 vs. baseline (B); ** p <0.001 vs. LPA or EGF alone.

We next examined another further downstream functional response: ERK phosphorylation. Phosphorylation of this key enzyme has been observed in response to LPA_1–3_ receptor activation [29–32]. As shown in Fig 7, activation of any of the three receptors studied was able to activate ERK as reflected by its phosphorylation state. The magnitude of the response was somewhat different, with that induced by LPA_1_ receptors greater and with a more prolonged duration than that induced by LPA_3_ receptor activation and that due to LPA_2_ activation was clearly smaller and lesser in duration (Fig 7). Elegant work by Ullrich and coworkers has shown that many GPCRs, including LPA receptors (subtype(s) not defined) can transactivate EGF receptors, through sequential metalloproteinase activation and HB-EGF shedding and that joint signaling through GPCRs and the EGF tyrosine kinase activity participates in some of the actions ([33] reviewed in [34–37]). Previously, we showed that activation LPA_1_ receptors induce Akt/PKB phosphorylation through the previously-mentioned EGF receptor transactivation process [12]. In the present experiments, the possible role of EGF receptor transactivation was evaluated, by inhibiting the EGF receptor kinase with the selective tyrphostin, AG1478 [38]. As presented in Fig 6, both LPA (1 μM) and EGF (100 ng/ml) increase ERK phosphorylation (~ 2- and ~ 4-fold, respectively). AG1478 clearly diminished the effect of both growth factors and, in some cases, to below the baseline signal (Fig 8). The baseline phospho-ERK signal was very low and AG1478 either did not alter it (cells expressing LPA_1_ receptors) or decrease it (cells overexpressing LPA_2_ (statistically insignificant) and LPA_3_ receptors (statistically significant) (“Fig D in S1 File”).

**Fig 9:**
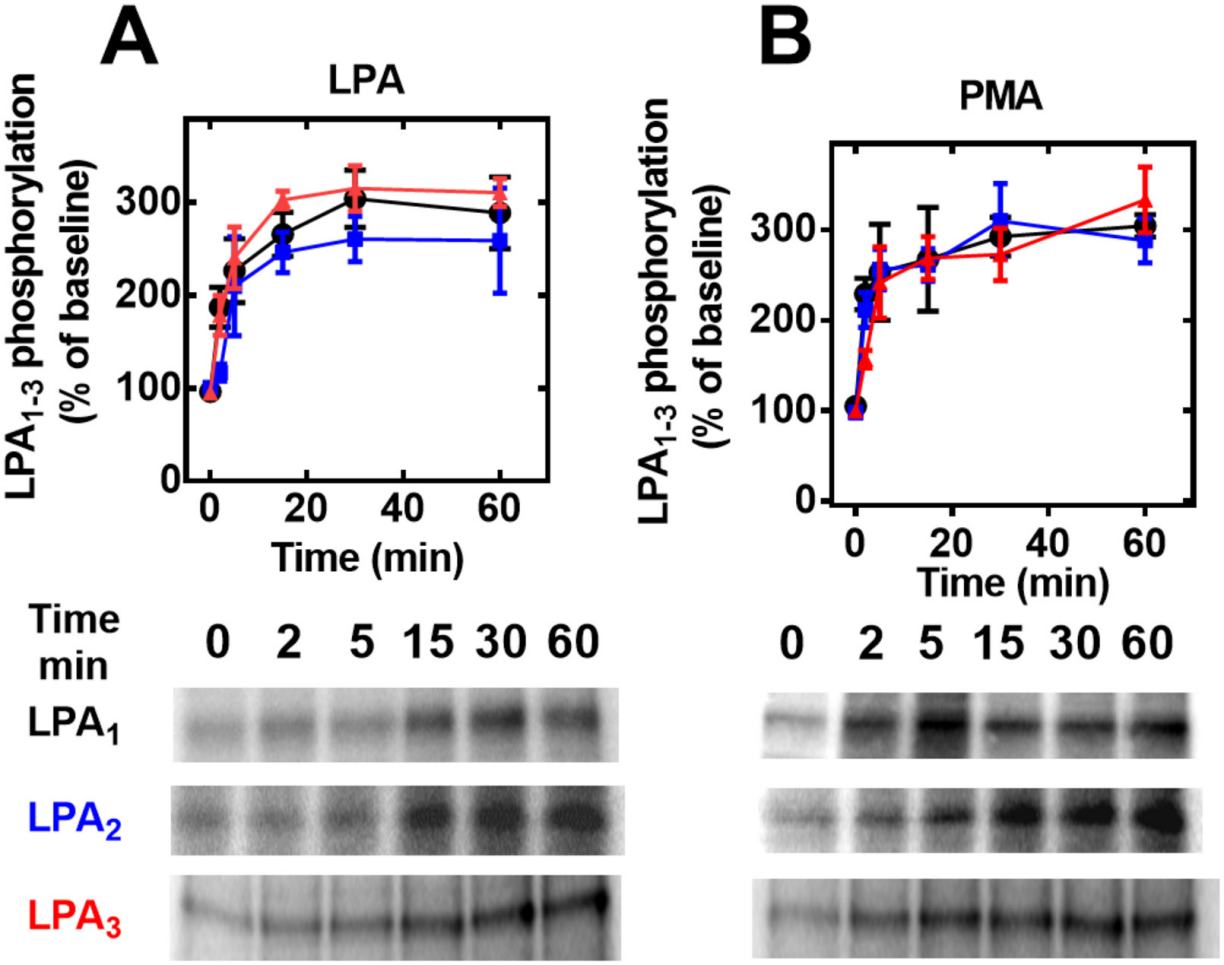
Time-courses of the effects of LPA and PMA on LPA_1–3_ receptor phosphorylation. Cells overexpressing LPA_1_ (black, circles), LPA_2_ (blue, squares) or LPA_3_ (red, triangles) receptors were incubated for the times indicated in the presence of 1 μM LPA (Panel A) or 1 μM PMA (Panel B). Plotted are the percentage of baseline phosphorylations as mean ± S. E. M. of 4–5 experiments using different cell preparations. Representative autoradiographs are presented for the different receptor subtypes.

**Fig 10:**
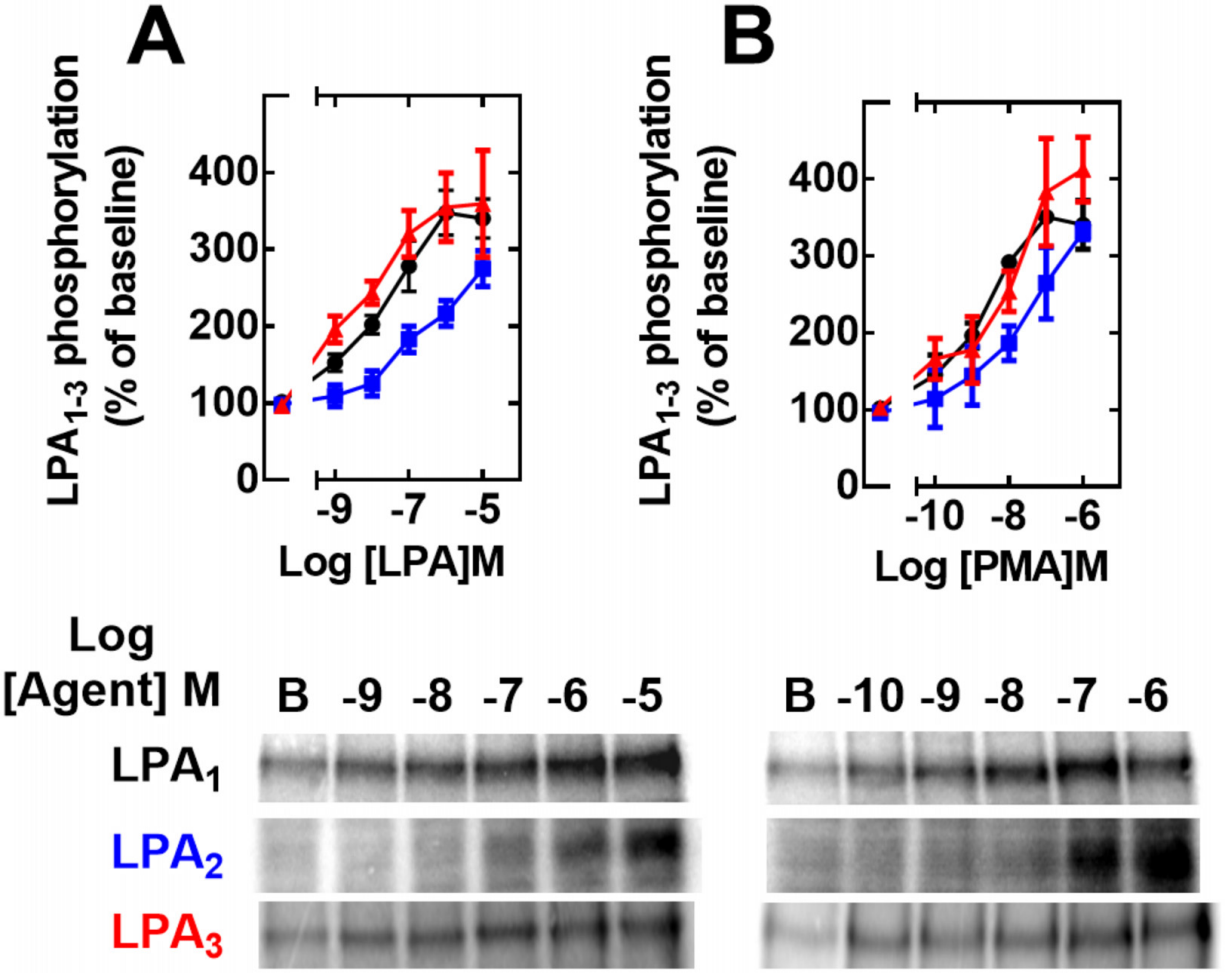
Concentration-response curves to LPA and PMA on LPA_1–3_ receptor phosphorylation. Cells overexpressing LPA_1_ (black, circles), LPA_2_ (blue, squares) or LPA_3_ (red, triangles) receptors were incubated for 15 min in the presence of different concentrations of LPA (Panel A) or PMA (Panel B). Plotted are the percentage of baseline phosphorylations as mean ± S. E. M. of 4–5 experiments using different cell preparations. Representative autoradiographs are presented for the different receptor subtypes.

Agonist- and PMA-induced receptor phosphorylation was examined next. Data showed that the three LPA receptors studied are phosphoproteins whose phosphorylation states are increased by LPA and the active phorbol ester, PMA (Figs 9 and 10). The time-course of the effect of 1 μM LPA (Fig 9, panel A) showed that in cells expressing any of the receptors studied, the agonist increased receptor phosphorylation, and this reached its maximum during the first 15 min and remained at the same level for up to 60 min. The effect of 1 μM PMA (Fig 9, panel B) took place faster than that of the agonist, reaching its maximum during the first 5 min and remaining at a plateau during the time studied (60 min). Interestingly, the relative magnitudes (percentage of baseline labeling) and temporal patterns were similar for all three receptor subtypes, although in some experiments LPA_2_ receptor phosphorylation was slightly delayed. The concentration-response curves to LPA and PMA are presented in Fig 10 (panels A and B, respectively). It can be observed that the curves for LPA and PMA were very similar for LPA_1_- and LPA_3_-overexpressing cells; saturation was obtained at ~ 1 μM LPA (EC_50_ 10–30 nM) and 100 nM PMA (EC_50_ ~ 3–10 nM) (Fig 10). In the studies utilizing cells that overexpress LPA_2_ receptors, no clear saturation was obtained for either LPA or PMA (Fig 10; panels A and B, respectively); under these conditions EC_50_ values could not be estimated, but the concentration-response curves exhibited a clear shift to higher concentrations as compared with those studying the remaining receptor subtypes.

**Fig 11:**
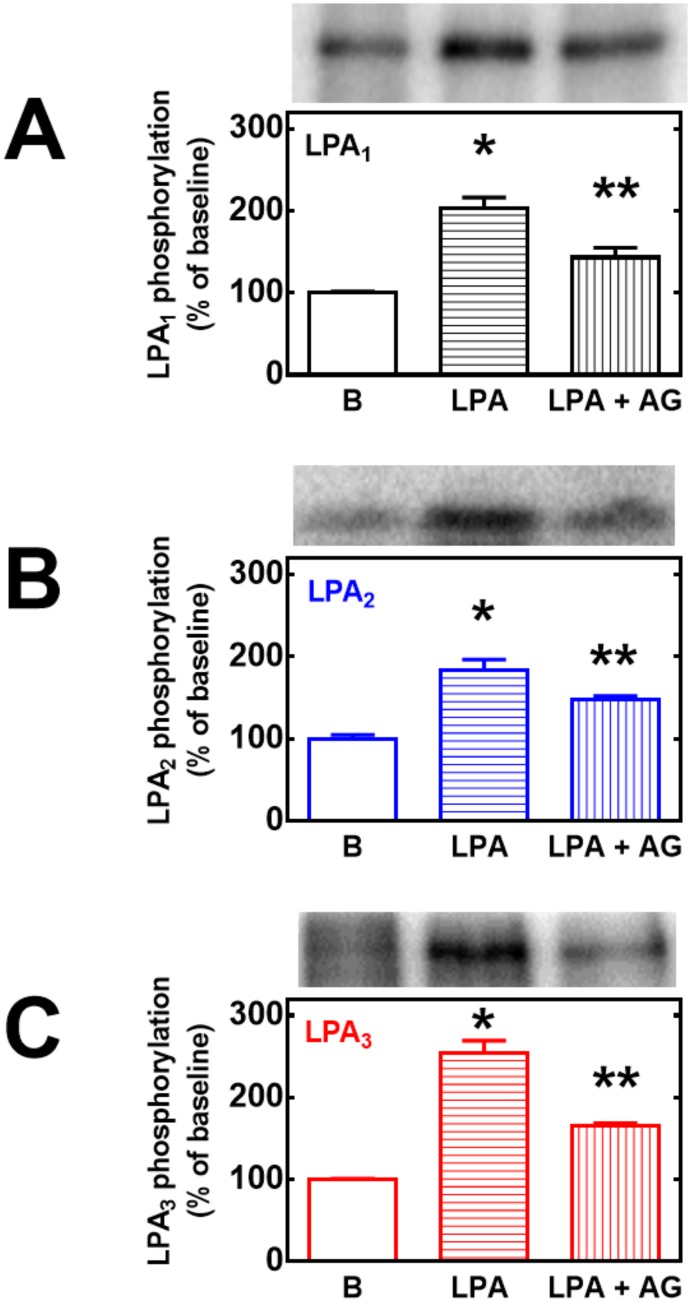
Role of EGF receptor transactivation on LPA-induced LPA_1–3_ receptor phosphorylation. Cells overexpressing LPA_1_ (panel A, black bars), LPA_2_ (panel B, blue bars) or LPA_3_ (panel C, red bars) receptors were preincubated for 30 min in the absence or presence of 10 μM AG1478 (+AG) and then incubated for 15 min in the absence or presence of 1 μM LPA. Plotted are the percentage of baseline (B) phosphorylations as mean ± S. E. M. of 4–5 experiments using different cell preparations. Representative autoradiographs are presented on the top of the figures for the different receptor subtypes. p < 0.001 vs. baseline; p < 0.05 vs. baseline and vs. LPA alone.

**Fig 12:**
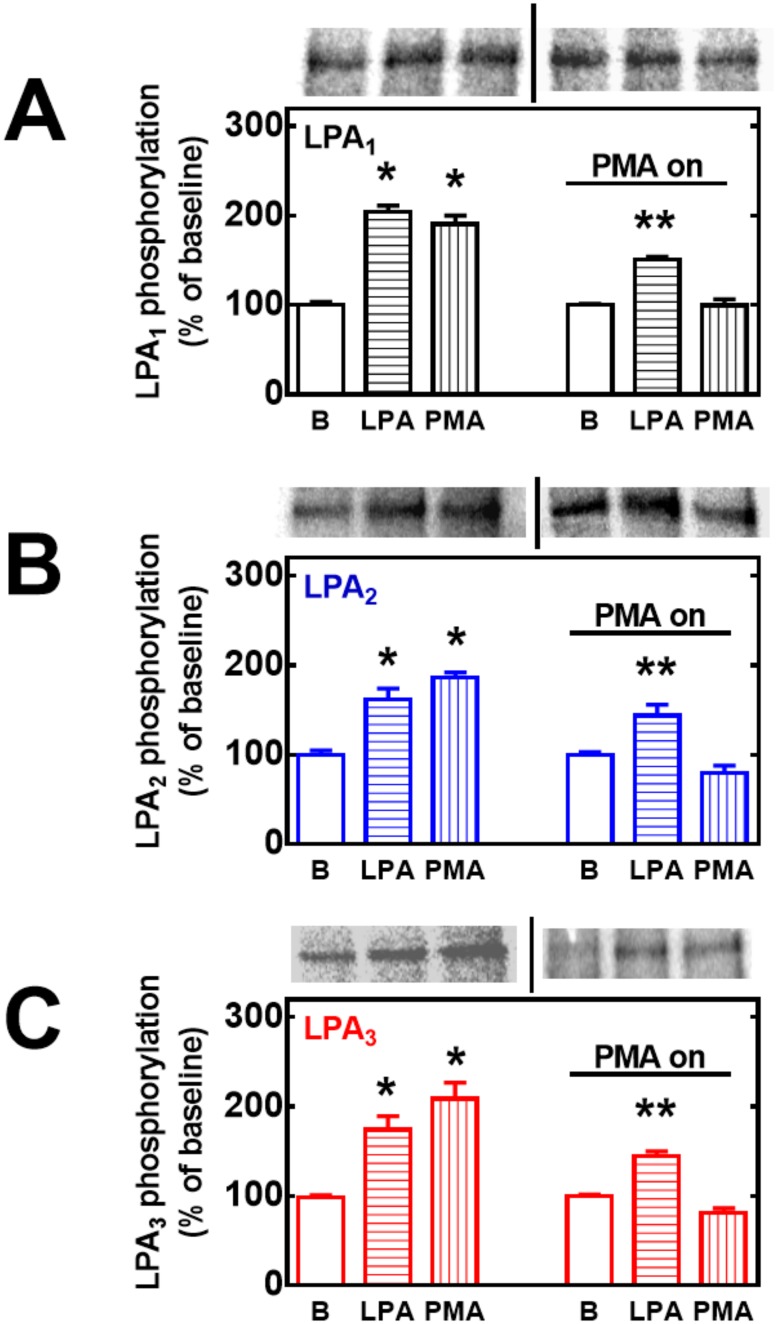
Role of PKC down-regulation on LPA- and PMA-induced LPA_1–3_ receptor phosphorylation. Cells overexpressing LPA_1_ (panel A, black bars), LPA_2_ (panel B, blue bars) or LPA_3_ (panel C, red bars) receptors were incubated in the absence or presence of 1 μM PMA overnight, washed and subjected to the receptor phosphorylation protocol. Cells were incubated for 15 min in the absence or presence of 1 μM LPA or 1 μM PMA. Plotted are the percentage of baseline (B) phosphorylations as mean ± S. E. M. of 3–4 experiments using different cell preparations. Representative autoradiographs separated by vertical lines are presented on the top of the figures for the different receptor subtypes. p < 0.001 vs. baseline; ** p < 0.05 vs. baseline.

**Fig 13:**
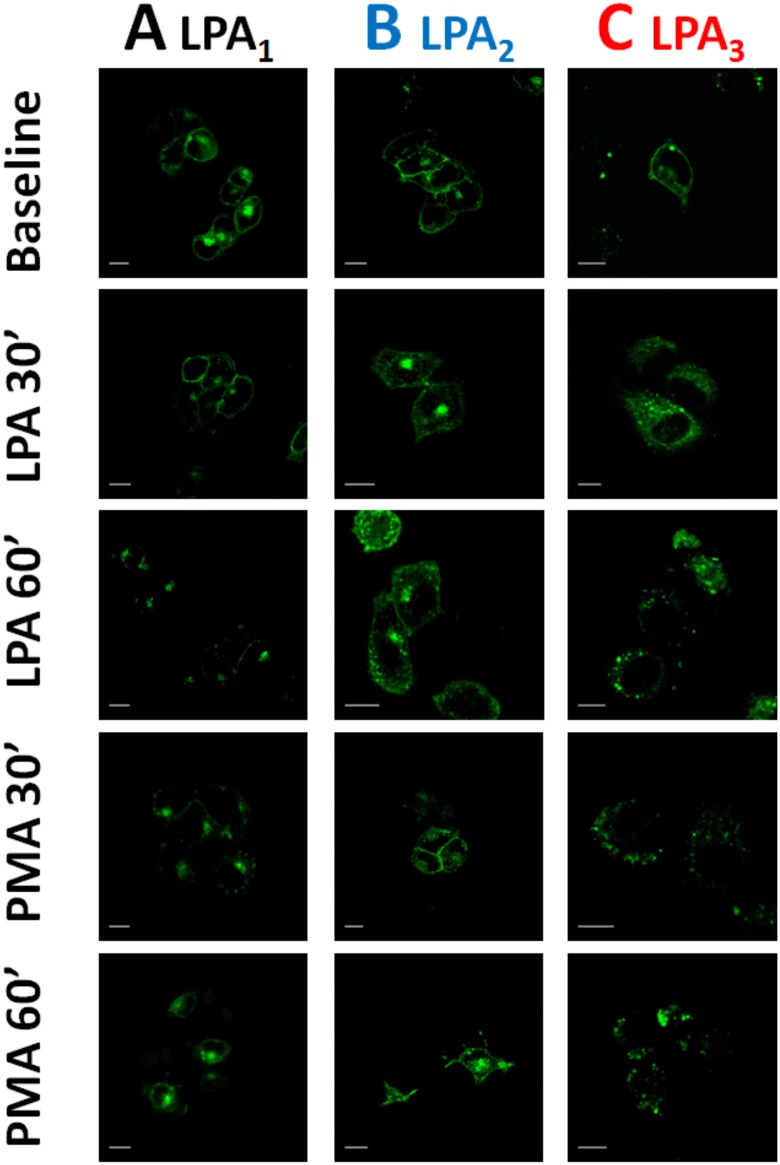
Images of the effects of LPA and PMA on LPA_1–3_ receptor internalization. Fluorescent confocal images of cells overexpressing LPA_1_ (column A), LPA_2_ (column B) or LPA_3_ (column C) receptors were incubated in the absence of any agent (Baseline) or for 30 or 60 min in the presence of 1 μM LPA or 1 μM PMA. Images are representative of data of 3–4 experiments using different cell preparations. Bars 15 μm.

**Fig 14:**
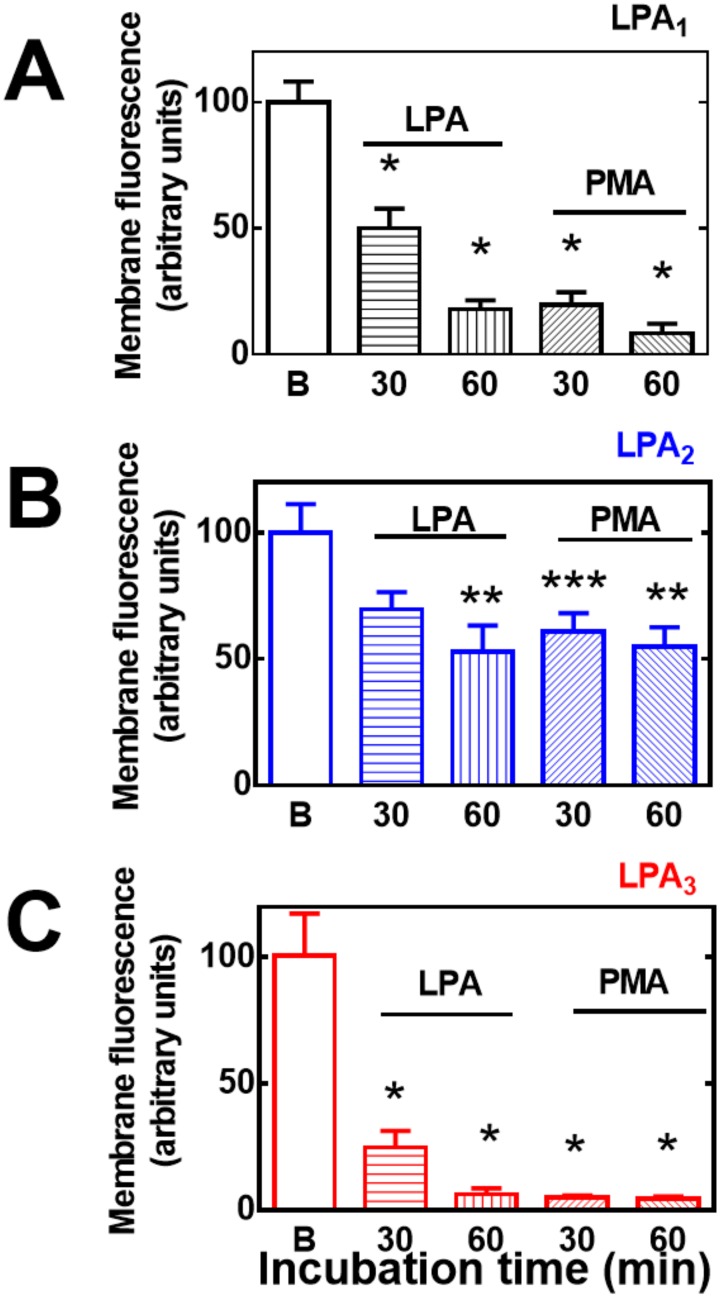
Effects of LPA and PMA on LPA_1–3_ receptor internalization. Cells overexpressing LPA_1_ (panel A), LPA_2_ (panel B) or LPA_3_ (Panel C) receptors were incubated for 30 or 60 min in the presence of 1 μM LPA or 1 μM PMA. Plotted is membrane-associated fluorescence (arbitrary units) as the mean ± S. E. M. of 5 different fields of 3–4 experiments using different cell preparations. * p <0.001 vs. baseline (B), ** p < 0.01 vs. baseline (B), *** p < 0.05 vs. baseline (B).

**Fig 15:**
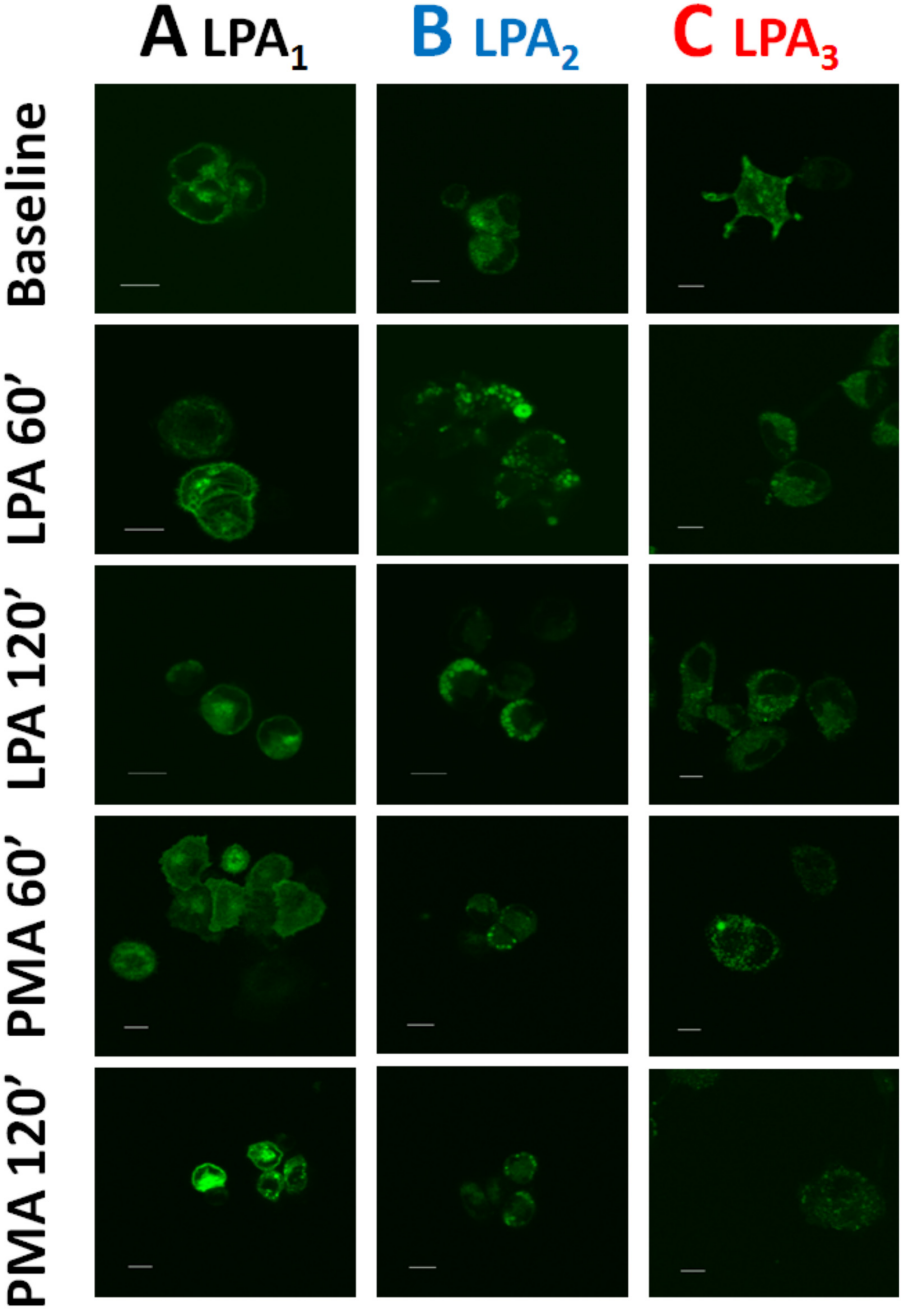
Images of the effects of LPA and PMA on LPA_1–3_ receptor internalization (60 and 120 min). Fluorescent confocal images of cells overexpressing LPA_1_ (column A), LPA_2_ (column B) or LPA_3_ (column C) receptors. Cells were incubated in the absence of any agent (Baseline), for 10 min in the presence of 1 μM LPA, or for 2 min in the presence of 1 μM PMA. After this incubation cells were extensively washed and further incubated for the times indicated. Images are representative of data of 3 experiments using different cell preparations. Bars 10 μm.

**Fig 16:**
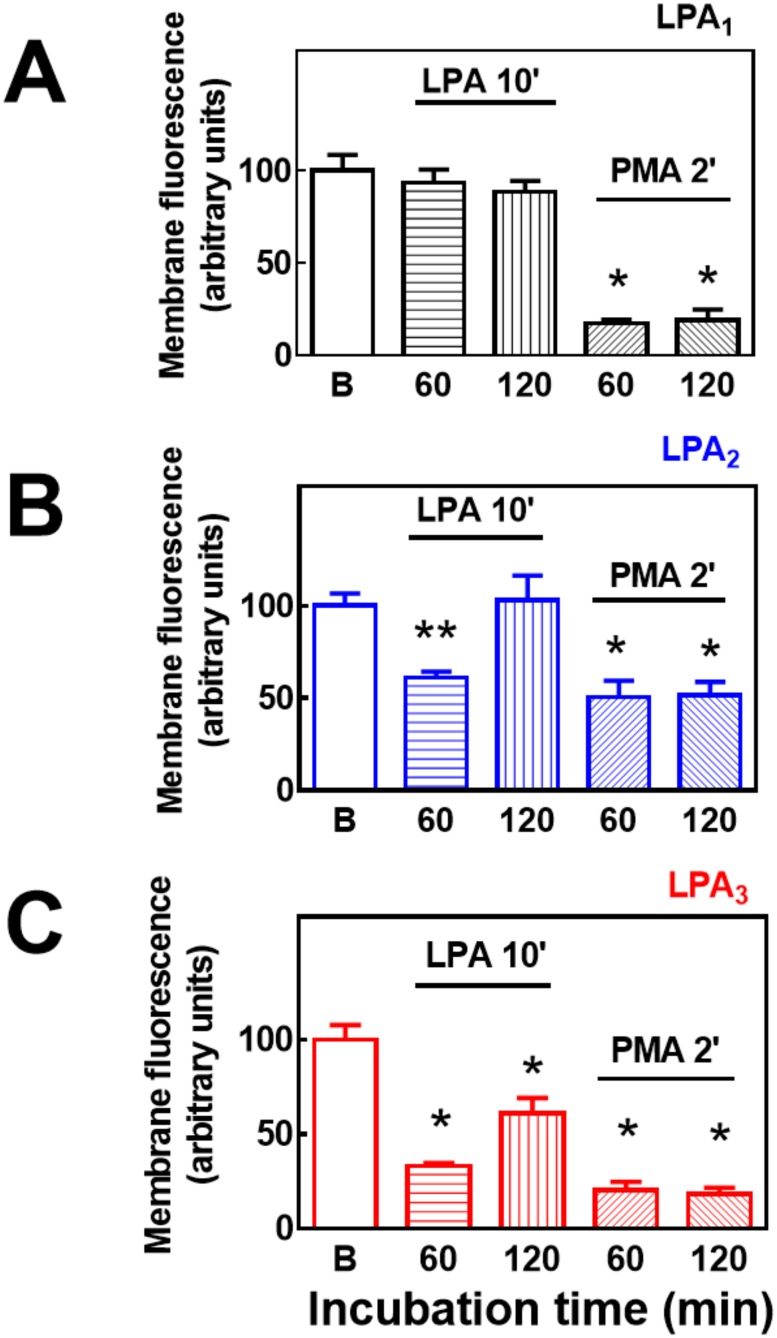
Effects of LPA and PMA on LPA_1–3_ receptor internalization (60 and 120 min). Cells overexpressing LPA_1_ (panel A), LPA_2_ (panel B) or LPA_3_ (Panel C) receptors were incubated in the absence of any agent (Baseline), for 10 min in the presence of 1 μM LPA, or for 2 min in the presence of 1 μM PMA. After this incubation cells were extensively washed and further incubated for the times indicated (60 or 120 min) Plotted is membrane-associated fluorescence (arbitrary units) as the mean ± S. E. M. of 5 different fields of 3 experiments using different cell preparations. * p <0.001 vs. baseline (B), ** p < 0.01 vs. baseline (B), *** p < 0.05 vs. baseline (B).

It has been observed that EGF receptor transactivation plays a role in the phosphorylation of some GPCRs, such as the α_1B_-adrenergic receptor [39–42]. As shown in Fig 11, this was also the case in agonist-induced LPA_1–3_ receptor phosphorylation, i. e., the EGF receptor tyrosine kinase inhibitor, AG1478, markedly reduced (but did not abolish) LPA-induced phosphorylation of the three receptor subtypes studied. The inhibitor by itself did not alter basal receptor phosphorylation (data not shown). As already mentioned, prolonged treatment (over-night) with 1 μM PMA markedly down-regulates conventional and novel PKC isoforms [14, 28]. Consistent with previous data on LPA_1_ receptors [14], this treatment markedly reduced or abolished PMA-induced LPA_1–3_ phosphorylation but did not alter LPA-induced receptor phosphorylation (Fig 12). GPCR phosphorylation appears to be associated with receptor internalization. Current ideas suggest that GPCR phosphorylation increases the receptor’s affinity for β-arrestins, which associates with clathrin favoring the formation of receptor-enriched coated pits, triggering internalization [43–46]. As depicted in the confocal images presented as in Fig 13, fluorescence (i. e. the eGFP-tagged receptors) was present in both, the plasma membrane and intracellular vesicles. Treatment with LPA or PMA clearly altered receptor distribution, markedly decreasing fluorescence at the plasma membrane level and increasing that in intracellular vesicles. Such changes were clearly observed only after 20–30 min indicating that desensitization precedes internalization. Differences were observed among the LPA receptor subtypes after continuous exposure to these agents for 30 or 60 min. The decrease in fluorescence at the plasma membrane was much less intense in LPA_2_-overexpressing cells as compared with that in those overexpressing the other subtypes (Fig 13) (see also images overlapping fluorescence and differential interference contrast in “Fig E in S1 File”); quantitative analysis also clearly evidenced this (Fig 14). Differences were also observed in the morphology (small punctuated or large vesicles) and localization (concentrated in perinuclear region or distributed in the whole cell) of the internalized fluorescence both among the distinct receptors studied and also depending on the stimulus. Analysis of such differences will require a systematic work with different approaches. In an effort to get further insight into the receptor traffic dynamics, images were obtained in cells treated with 1 μM LPA (10 min), exhaustively washed, and further incubated to complete 60 or 120 min of incubation (Fig 15). It was observed that under these conditions fluorescence recovered rapidly and completely at the membrane level in cells expressing LPA_1_ receptors; slowly in cells expressing LPA_2_ receptors, and only partially and also slowly in cells expressing LPA_3_ receptors (Fig 15, see also “Fig F in S1 File”); quantitative analysis is presented in Fig 16. Fluorescence recovery at the plasma membrane could represent, receptor recycling, incorporation of receptors previously present in vesicles and newly synthesized ones, and, more likely a mixture of all these. Distinction among such processes will also require a systematic work with different approaches.

## Discussion

In the present work, we comparatively analyzed LPA _1–3_ receptor signaling and desensitization in the same cellular model, C9 cells. The actions and mechanisms of action of these receptors have been extensively studied but data on their phosphorylation are scarce. LPA-, PMA- and angiotensin II-induced LPA_1_ receptor phosphorylation has been observed [4, 10–14] and in an elegant work, Kuriyama et al. reported that LPA signaling is required, during *Xenopus* development, for neural crest migration and that such action involves LPA_2_ receptor phosphorylation at serine 324 [47]. We are not aware of any publication describing LPA_3_ phosphorylation. In the present work eGFP tagged-LPA receptors were employed because it allowed us to use fluorescence confocal microscopy to follow the receptors’ locations within the cells and to immunoprecipitate the receptors, using anti-eGFP antisera. This is a common strategy and the eGFP itself does not appear to be a phosphorylation substrate [11, 48, 49]. Our data clearly showed that the three receptors studied, i. e., LPA_1–3_, are phosphoproteins whose phosphorylation state is modulated by the natural agonist, LPA, and by pharmacological activation of PKC by PMA. These data are consistent with *in silico* analysis, which revealed potential phosphorylation sites in the structure of these three receptors [4]. Alignment of these receptors’ intracellular loops 2 and 3 and of the carboxyl termini showed that some of these putative phosphorylation sites are conserved but many of them are not [4]. Interestingly, many of the putative sites for GRKs were located in the carboxyl termini, whereas those for PKC were mainly in the third intracellular loop; however, sites for these protein kinase families were located both in intracellular loops and in the carboxyl tail [4]. Putative phosphorylation sites for other protein kinases were also present in these receptors; these include sites for protein kinase A, Akt/PKB, calcium/calmodulin protein kinase, AMP-dependent protein kinase, and receptor and non-receptor tyrosine kinases [4]. Future structural work will be required to determine the site(s) that are real target(s) of theses protein kinases *in cellulo* and the functional repercussion of such covalent modifications. The importance of studying receptor phosphorylation sites is multiple. There is evidence that GPCR phosphorylation is important in favoring their interaction with β-arrestins, and that such interaction participates in both receptor internalization and signaling ([50–52] and references therein, see also [53, 54]). Evidence indicating that internalized GPCRs continue signaling in endosomes is accumulating [55–58] and this appears to vary among different receptor types [58]. Additionally, receptors are phosphorylated in different residues depending on the stimulus (i. e., the protein kinases involved, such as GRKs, second messenger-activated kinases and others) and the cell context (likely reflecting the repertoire of protein kinases and other interacting proteins expressed). This has been denominated the “phosphorylation bar code” and has been suggested to define the interaction of receptors with other proteins and hence the internalization processes involved, the receptors’ fate (recycling/ degradation) and their endosomal signaling [19, 23, 59]. Our group has recently provided evidence that a GPCR, the α_1B_-adrenergic receptor, interacts with different proteins and internalizes into distinct endosomal compartments during homologous and heterologous desensitizations [24]. At this point, little is known on these aspects for LPA receptors and for many other GPCRs.

It is clear from our data that the phosphorylation of the LPA receptors studied and its functional repercussion differ among subtypes and the triggering process, i. e. agonist-stimulated vs. PMA action. PKC appears to be involved in PMA action, as evidenced by the use of the inhibitor, bisindolylmaleimide I, and PKC down-regulation (overnight incubation with the active phorbol ester). In contrast, PKC does not appear to play a significant role in agonist-induced desensitization. Work by Iacovelli and coworkers [45] have shown that in FRTL-5 cells, which endogenously express LPA_1–3_ receptors, LPA markedly inhibits forskolin-stimulated cyclic AMP accumulation and increases ERK 1/2 phosphorylation; these effects were attenuated by overexpression of GRK2 or β-arrestin 1[45]. Similarly, it has been observed that GRK2 is required for agonist-induced desensitization of LPA_1_ and LPA_2_ receptors transfected into HEK293 cells [60]. With these data and our present findings, it appears probable that major roles might be played by GRKs (likely GRK2) and β-arrestin in homologous desensitization/ phosphorylation.

LPA-induced desensitization of the three studied receptors was characterized by a decreased sensitivity to the agonist, as evidenced by a shift to the right of the concentration-response curves; this was less pronounced in cells expressing the LPA_2_ subtype. In contrast, PKC activation with PMA resulted in a much diminished response even to high LPA concentrations. In addition, the effect of LPA was reversible, whereas that of PMA was not, during the times explored. I should be considered that LPA interacts with GPCRs at the external surface of the plasma membrane, is rapidly metabolized and can be removed by extensive washing whereas PMA acts intracellularly (mainly on PKC), is very lipophilic and its complete removal by washing is rather unlikely. This might explain some of the differences observed. However, angiotensin II is also able to induce LPA_1_ phosphorylation and desensitization, with the involvement of PKC [11, 12]. Work in progress in our laboratory indicates that angiotensin II-induced LPA_1_ desensitization is not readily reversible after extensive washing. These data suggest that the differences among the mechanisms involved in these desensitization processes likely play also a role. Agonist-induced LPA_1_ internalization has been reported by several groups [10, 11, 60–64] and appears to involve membrane cholesterol, β-arrestin, dynamin, and Rab 5 [62–64]. A cluster of serine residues in the receptor’s carboxyl terminus seems to be required for β-arrestin translocation to the plasma membrane [64]. Interestingly, it has been observed that β-arrestin is not required for PMA-induced LPA_1_ receptor internalization [64], which further emphasizes the differences between LPA-mediated processes and those induced by pharmacological activation of PKC. Agonist-triggered internalization of LPA_2_ receptors has also been studied [60, 65]. In one of these works clear agonist-induced receptor internalization was observed [60] whereas in the other, internalization was slow and very limited in magnitude [65]; marked differences in the experimental conditions (i. e., cell types, conditions for LPA exposure and in the detection of membrane receptors) might explain the disparate results. Interestingly, LPA_2_ receptors are key elements in the formation of the macromolecular complexes that mediate LPA gradient sensing in fibroblasts [65]. In our work, we consistently observed that LPA- and PMA-induced LPA_2_ receptor internalizations were of lesser magnitude than those observed with the remaining subtypes studied. Similarly, we observed that phosphorylation of the LPA_2_ receptor subtype required higher concentrations of agonist or PMA. A link between receptor phosphorylation and internalization might exist, but no causal relationship can be defined at this point and, as mentioned, different approaches will be required. To the best of our knowledge internalization of LPA_3_ receptors has not been previously reported.

As already mentioned, many GPCRs, including LPA receptors can transactivate EGF receptors, an effect important for many of the actions of this lysophospholipid [34–37]. Numerous studies have highlighted this action for LPA_1_ receptors (see for example [12, 31, 66]) and there is evidence that LPA_2_ [67–69] and LPA_3_ [30, 32] receptors also employ in their signaling this transactivation process. EGF receptors transactivation is a complex process that can or cannot involve, changes in intracellular calcium, metalloproteinase activation, shedding of membrane-bound EGF activators (TGF-α, HB-EGF, amphiregulin, betacellulin, and epiregulin, among others) non-receptor tyrosine kinases (such as Src and Pyk), second messenger-activated kinases (such as PKC o PI3K) and other molecular elements. Two major processes have been defined. One of them involves the release of membrane-bound EGF ligands and autocrine/ paracrine cell communication, whereas the other takes place intracellularly without the release of a messenger. These processes are not mutually exclusive and can coexists in the same cell (see [34–37] and references therein).

Our data also showed that the ability to transactivate EGF receptors is shared by the three LPA receptors studied, and that such action is required for some actions (such as ERK phosphorylation and agonist-induced receptor phosphorylation). In a previous work, we observed that EGF induces LPA1 desensitization, consistent with LPA1-EGFR functional crosstalk [12]. Recent work has shown that antidepressants and LPA induce tyrosine phosphorylation of insulin-like growth factor receptors and insulin receptor substrate-1, involving LPA_1_ receptors and Src activation [66]. It is clear that further work is necessary to fully understand the regulation of LPA_1–3_ receptors; this has medical and biological importance, considering the many functions in which these lysophosphospholipid-activated receptors are involved and their roles in the pathogenesis of morbid entities.

## Supporting Information

S1 File Supplementary Figs A-F are included in this file. (PDF)
