# Supplementary material for: Phosphorylation and Internalization of Lysophosphatidic Acid Receptors LPA1, LPA2, and LPA3
Source: PLoS One. 2015 Oct 16;10(10):e0140583. doi: 10.1371/journal.pone.0140583 (PMC4608732; doi:10.1371/journal.pone.0140583)

SUPPLEMENTARY FIGURES

Fig A. Flow cytometry and confocal images of C9 cells overexpressing $LPA_{1\text{-}3}$ receptors fused to the enhanced green fluorescent protein. Flow cytometry profile of wild type (WT, yellow line) C9 cells and those overexpressing LPA_1 (black line), LPA_2 (blue line) and LPA_3 (red line) receptors. Confocal images (10X) of cells overexpressing $LPA_{1\text{-}3}$ receptors fused to the eGFP. Bars 15 µm.

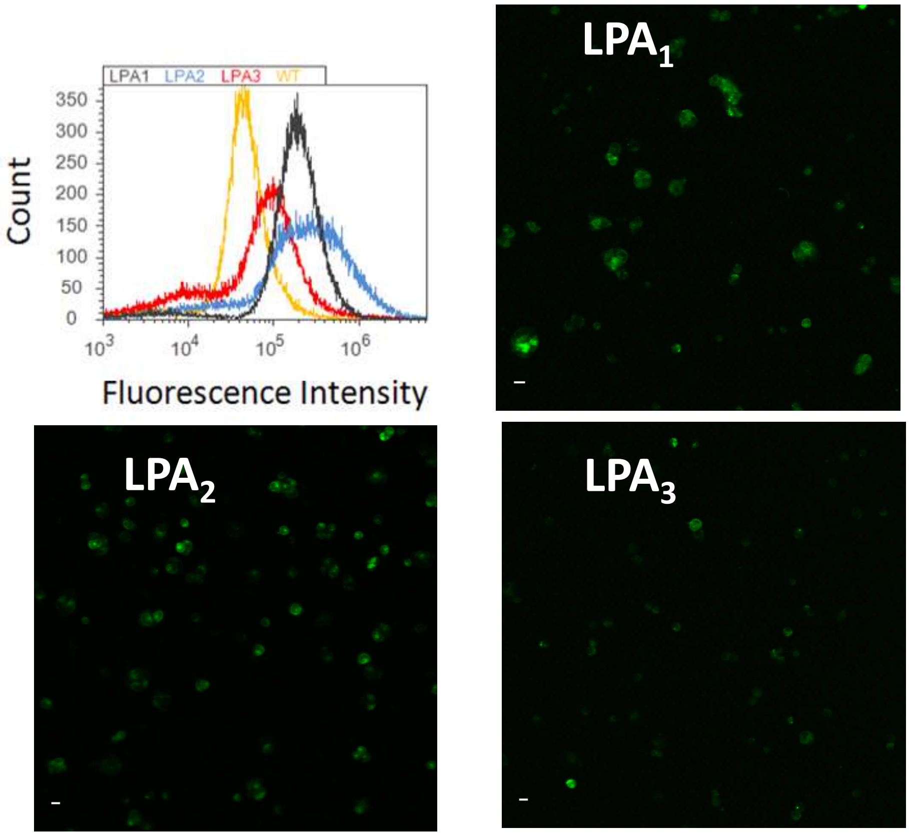

Fig B. Representative calcium tracings of wild type C9 cells and cells overexpressing $LPA_{1\text{-}3}$ receptors. Wild type cells (black lines, panel A) and those overexpressing LPA_1 (black lines, panel B), LPA_2 (blue lines, panel C) and LPA_3 (red lines, panel D) were preincubated for 2 min in the absence (tracings on the left) or presence (tracing on the right) of 1 µM PMA. After this incubation the cells were stimulated (arrows) with 1 µM LPA.

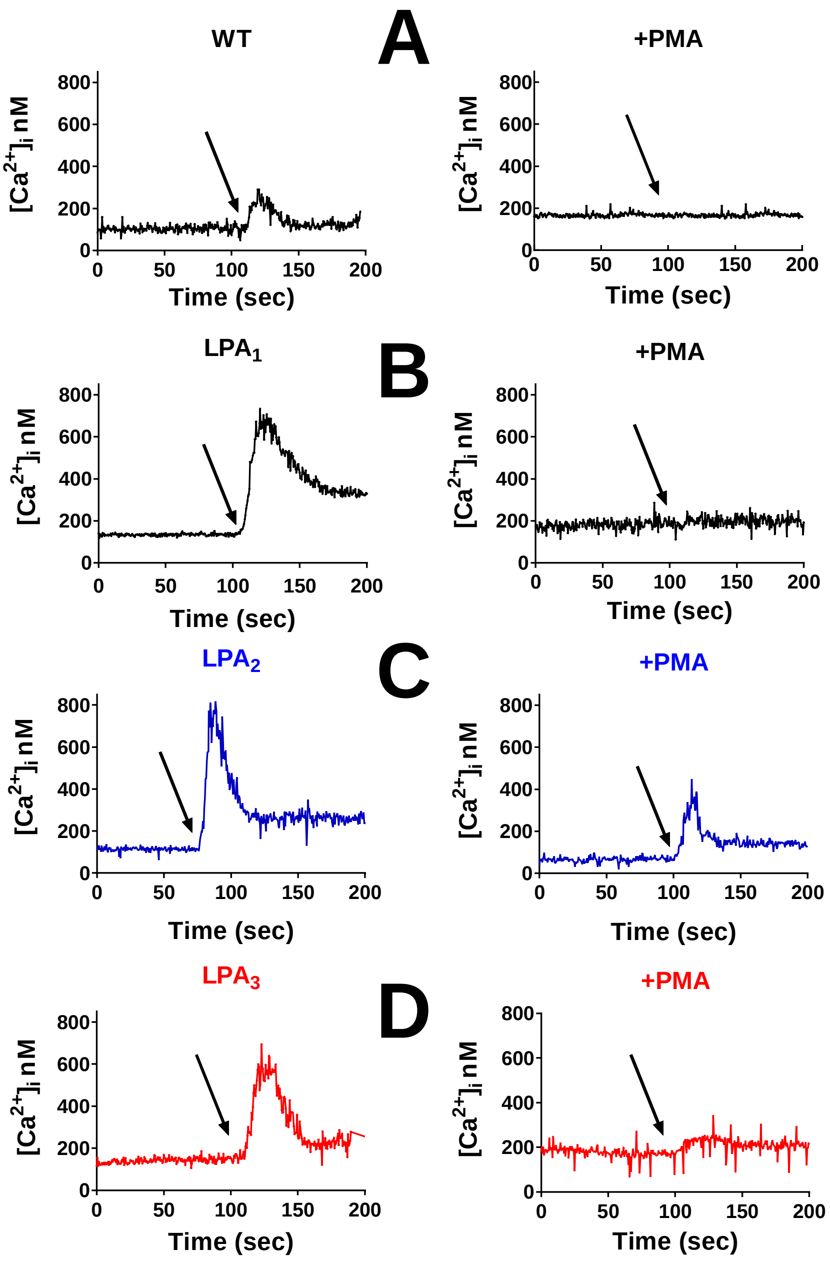

Fig C. Western blotting of cell extracts of cells overexpressing $LPA_{1\text{-}3}$ receptors. Cells overexpressing LPA_1, LPA_2, or LPA_3 receptors tagged with the eGFP were incubated for the times indicated in the presence of 1 µM LPA or 1 µM PMA. All incubations were carried out in the presence of 50 µM cycloheximide. After this incubations whole-cell extracts were obtained, subjected to SDS-PAGE and electrotransferred onto polyvinyliden fluoride membranes and subjected to Western blotting using monoclonal anti-GFP antibodies. Blots are representative of 3 experiments using different cell preparations.

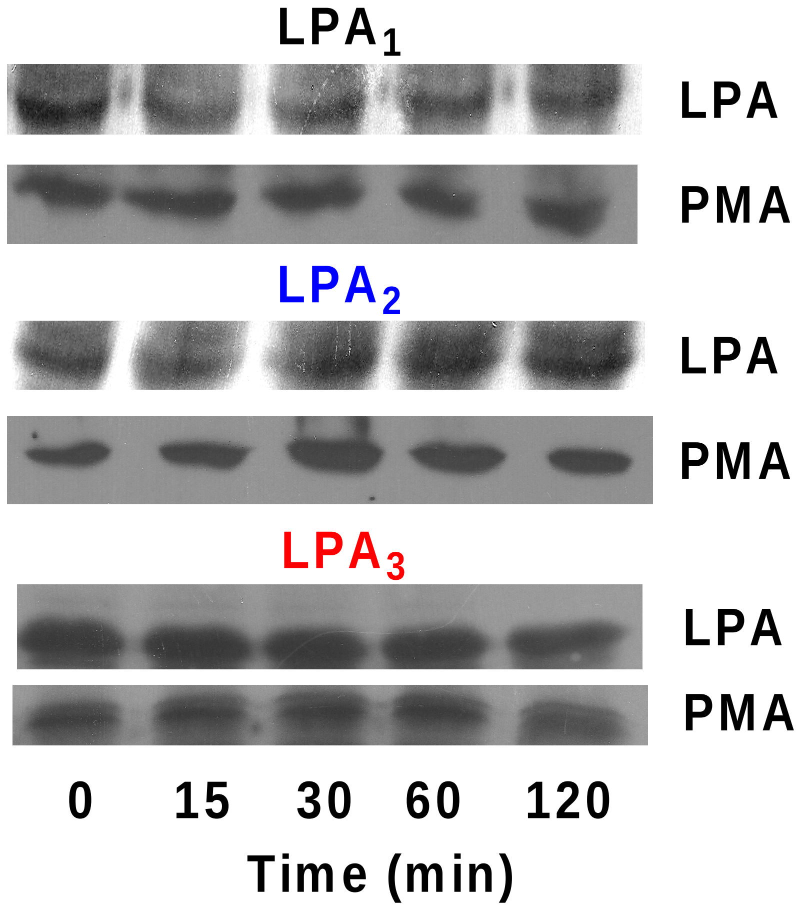

Fig D. Effect of AG1478 on baseline ERK 1/2 phosphorylation. Cells overexpressing LPA_1 (black bars, panel A; panel B), LPA_2 (blue bars, panel A; panel C) and LPA_3 (red bars, panel A; panel D) were preincubated for 30 min in the absence or presence of 10 µM AG 1478 (AG). In panel A plotted are the means $\pm$ S.E. M. of 3 data. *$p < 0.001$ vs. absence of AG 1478. The experimental data are shown in panel B (LPA_1-expressing cells), panel C (LPA_2-expressing cells) and panel D (LPA_3-expressing cells). Base line signal was very small; therefore, film was overexposed to allow detection.

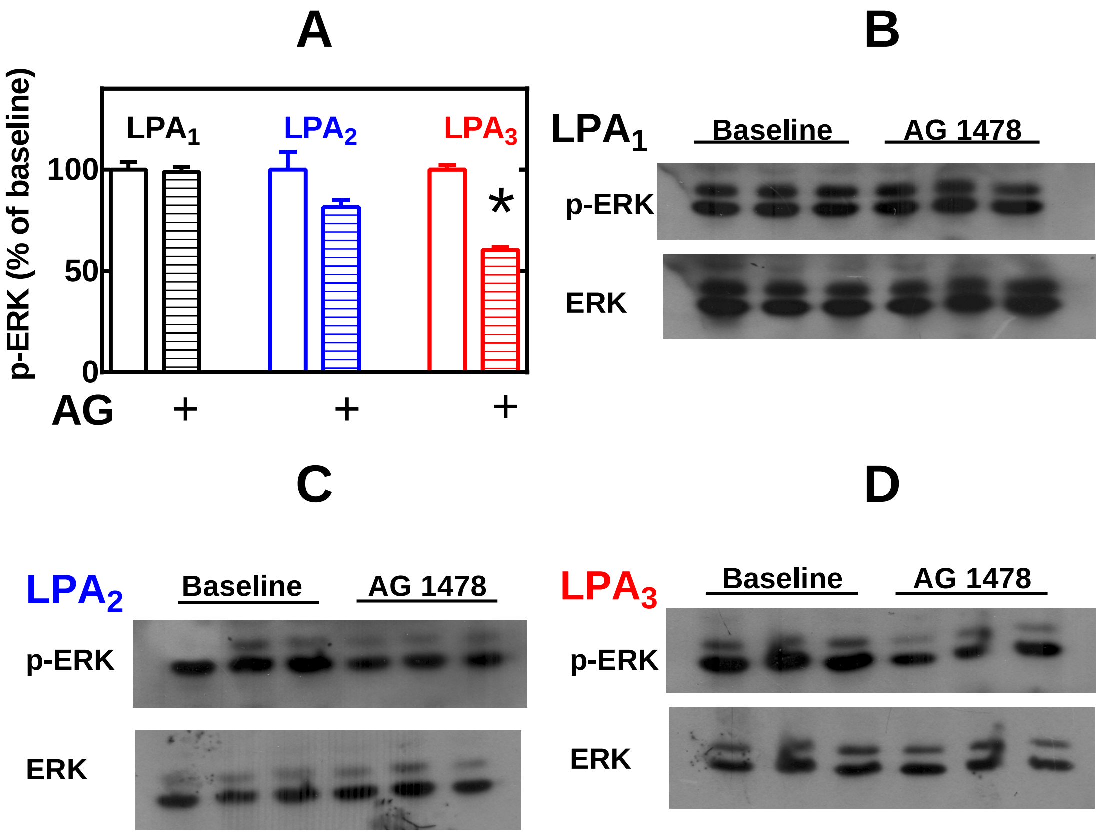

Fig E. Images of the effects of LPA and PMA on LPA_{1-3} receptor internalization. Overlapping of differential interphase contrast and fluorescent confocal images of cells overexpressing LPA_1 (column A), LPA_2 (column B) or LPA_3 (column C) receptors. Cells were incubated in the absence of any agent (Baseline) or for 30 or 60 min in the presence of 1 µM LPA or 1 µM PMA. Images are representative of data of 3-4 experiments using different cell preparations. Bars 15 µm.

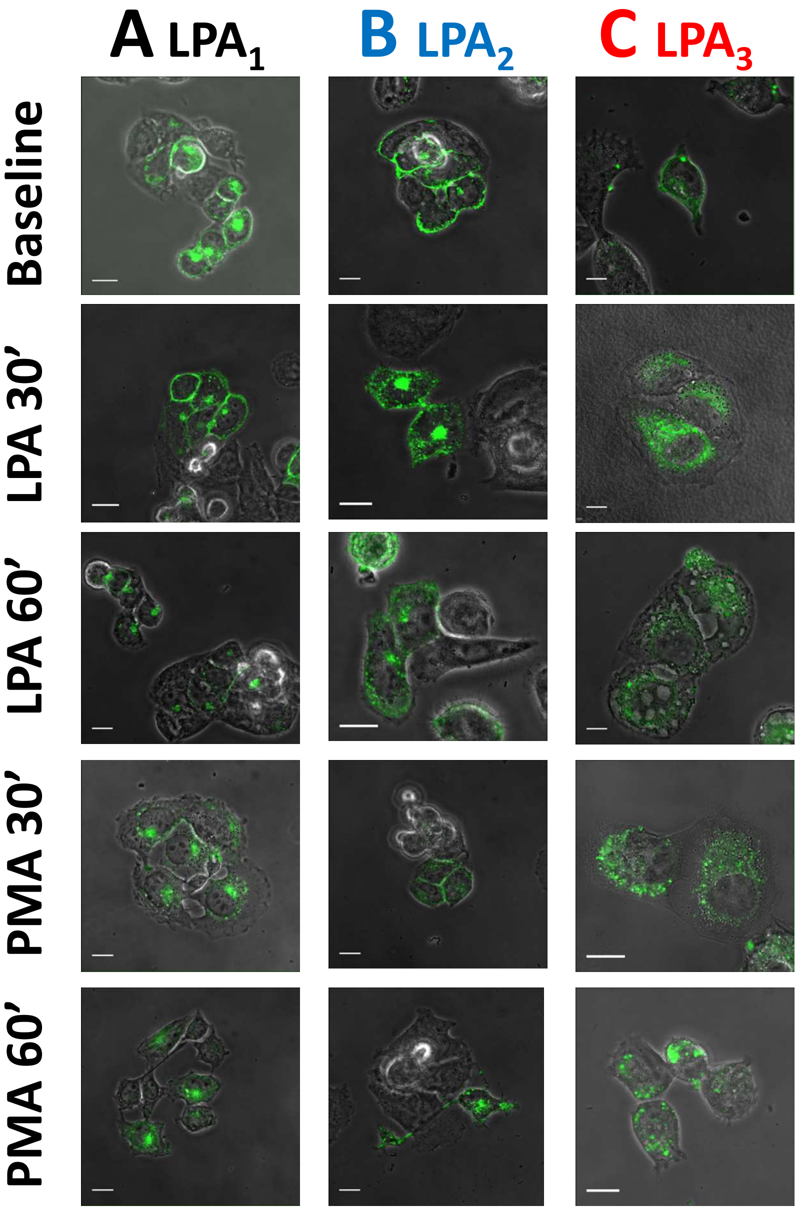

Fig F. Images of the effects of LPA and PMA on $LPA_{1\text{-}3}$ receptor internalization. Overlapping of differential interphase contrast and fluorescent confocal images of cells overexpressing LPA_1 (column A), LPA_2 (column B) or LPA_3 (column C) receptors. Cells were incubated in the absence of any agent (Baseline), for 10 min in the presence of 1 µM LPA, or for 2 min in the presence of 1 µM PMA. After this incubation cells were extensively washed and further incubated for the times indicated. Images are representative of data of 3-4 experiments using different cell preparations. Bars 10 µm.

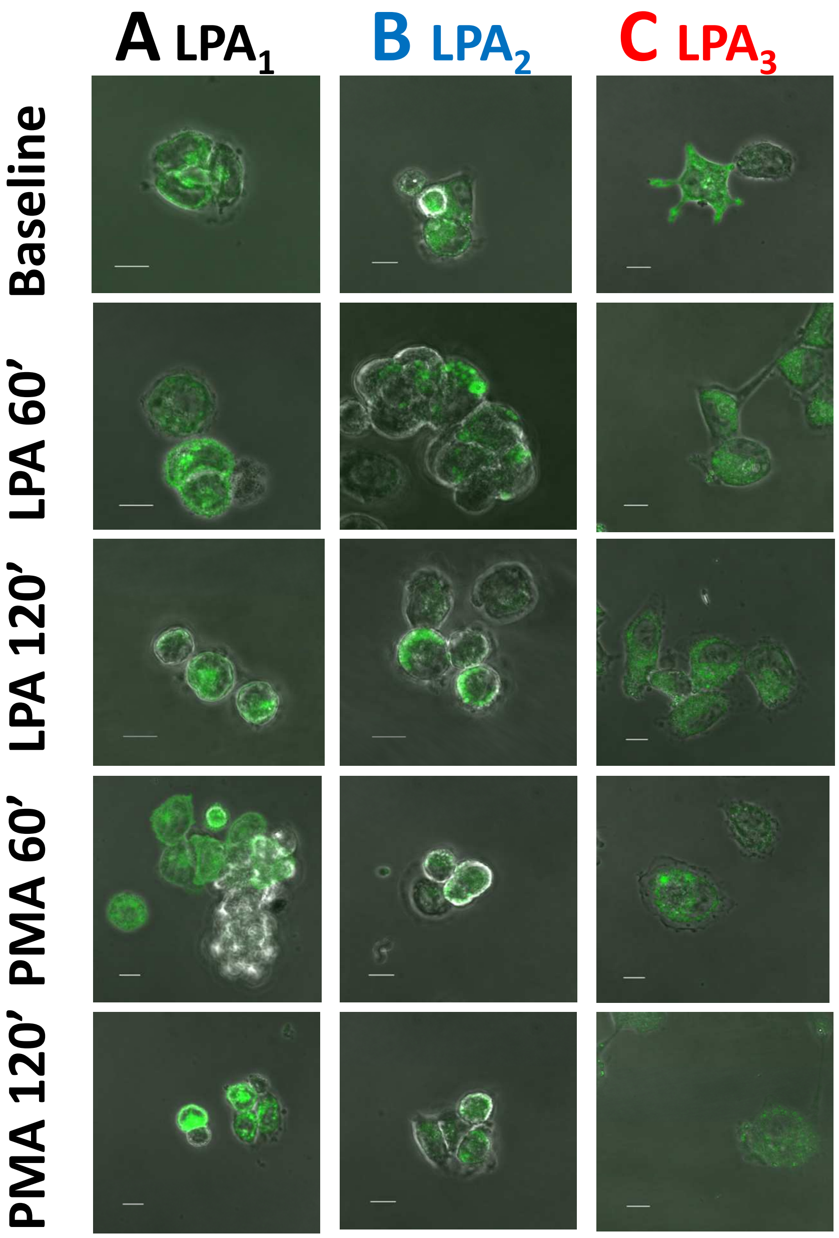

Supplement: S1 File — (PDF) [file pone.0140583.s001.pdf]
